# Pathway remodeling and adaptive evolution enable efficient co-utilization of glucose and xylose in *Escherichia coli*

**DOI:** 10.1128/mbio.01150-26

**Published:** 2026-07-14

**Authors:** Xiaoxu Tan, Yichun Hu, Kai Li, Wensi Meng, Kaiyu Gao, Qiaoyue Yang, Lingru Gong, Leilei Guo, Mengyao Hao, Hongxu Zhang, Yidong Liu, Zhaoqi Kang, Cuiqing Ma, Chao Gao, Qianjin Kang, Chuanjuan Lü, Ping Xu

**Affiliations:** 1 State Key Laboratory of Microbial Technology, Shandong University 214177, Qingdao, People’s Republic of China; 2 Shanghai Changing Biotechnology Co., Ltd, Shanghai, People’s Republic of China; 3 State Key Laboratory of Microbial Metabolism, Joint International Research Laboratory of Metabolic & Developmental Sciences, and School of Life Sciences & Biotechnology, Shanghai Jiao Tong University 553742, Shanghai, People’s Republic of China; 4 SJTU Asia-Pacific Graduate Institute (SJTU-APGI), Singapore, Singapore; The University of Oklahoma, Norman, Oklahoma, USA

**Keywords:** lignocellulosic hydrolysate, carbon catabolite repression, *Escherichia coli*, pyruvate, 2,3-butanediol

## Abstract

Efficient co-utilization of glucose and xylose is critical for microbial bioconversion of lignocellulosic hydrolysates. However, carbon catabolite repression prevents simultaneous sugar consumption in conventional industrial strains such as *Escherichia coli*. Here, we blocked the Embden−Meyerhof−Parnas and pentose phosphate pathways in *E. coli* MG1655 by deleting *pgi* and *gnd*, generating strain *E. coli* MD0 that metabolizes glucose exclusively via the Entner−Doudoroff pathway. Adaptive laboratory evolution yielded mutant *E. coli* MDE with superior glucose-xylose co-utilization, outperforming *E. coli* MG1655Δ*ptsG*. Genomic analysis identified *gntR* and *xylR* mutations as key contributors to this phenotype. Similar engineering in *Klebsiella oxytoca* also enhanced glucose and xylose co-utilization. Seven byproduct genes were deleted in *E. coli* MDE, and efficient production of pyruvate from straw hydrolysate was achieved by using the constructed strain *E. coli* MDE-6. Further introducing the *budRABC* operon in *E. coli* MDE-6 resulted in 2,3-butanediol generation from straw hydrolysate. This study establishes *E. coli* MDE as a robust chassis for lignocellulose biorefinery.

**IMPORTANCE:**

The inability of industrial microbes to co-utilize glucose and xylose severely limits lignocellulosic biomass valorization in bioproduction. Here, we combined pathway separation and adaptive laboratory evolution to develop an *Escherichia coli* mutant MDE capable of simultaneous glucose and xylose utilization. Strain *E. coli* MDE was engineered to efficiently produce pyruvate and 2,3-butanediol from straw hydrolysate and showed robust sugar co-utilization at various ratios. We identified two key beneficial mutations enabling this phenotype. When introduced into *Klebsiella oxytoca* alongside the pathway separation strategy, these mutations also conferred efficient glucose-xylose co-utilization. This study provides a generalizable strategy for engineering simultaneous sugar utilization in diverse microbial chassis.

## INTRODUCTION

Lignocellulosic biomass represents the most promising resource for the production of renewable biofuels and chemicals (1–3). Glucose and xylose are the two most abundant fermentable sugars in lignocellulosic hydrolysates, accounting for over 90% of the total utilizable carbohydrates (4). However, most industrial microorganisms prioritize glucose consumption over xylose due to carbon catabolite repression (CCR) (5), which results in inefficient utilization of carbon sources and reduced bioprocess productivity. Therefore, circumventing CCR and enhancing the co-utilization efficiency of glucose and xylose is critical for the cost-effective production of biochemicals from lignocellulosic hydrolysates (6).

*Escherichia coli* is a widely used model organism for the conversion of sugars into value-added biochemicals, possessing endogenous metabolic pathways for both glucose and xylose. Glucose is first phosphorylated to glucose-6-phosphate (G6P) by glucokinase (Glk) or the phosphoenolpyruvate:carbohydrate phosphotransferase system (PTS). G6P is then either isomerized to fructose-6-phosphate (F6P) by glucose-6-phosphate isomerase (Pgi) to enter the Embden−Meyerhof−Parnas (EMP) pathway or oxidized to ribulose 5-phosphate (Ru5P) by glucose-6-phosphate dehydrogenase (Zwf), 6-phosphogluconolactonase (Pgl), and 6-phosphogluconate dehydrogenase (Gnd) to enter the pentose phosphate (PPP) pathway. For xylose, it is first converted to xylulose-5-phosphate (X5P) by xylose isomerase (XylA) and xylulokinase (XylB). X5P is subsequently either directly metabolized via the PPP pathway or converted to glyceraldehyde-3-phosphate (G3P) and F6P by transaldolase and transketolase for degradation through the EMP pathway. Thus, the metabolic pathways of glucose and xylose in *E. coli* are highly overlapping, and both rely on the EMP and PPP pathways (7).

**Fig 1:**
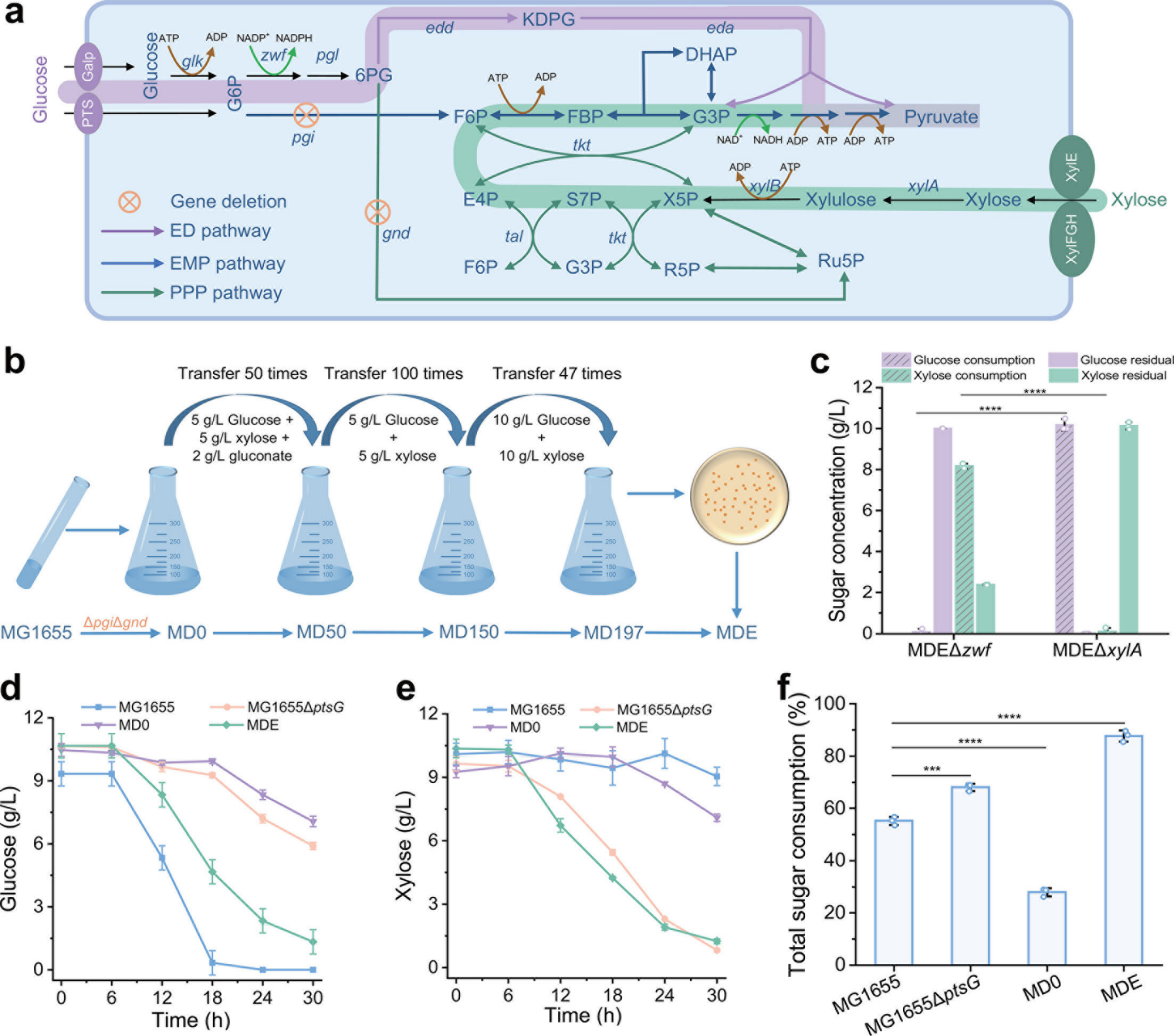
Design strategy to obtain the efficient glucose and xylose co-utilizing strain *E. coli* MDE. (**a**) Schematic illustration of glucose and xylose metabolism in *E. coli* MD0. PTS, phosphotransferase system; GalP, galactose permease; XylE, xylose transporter; XylFGH, xylose ABC transporters; *glk*, glucokinase coding gene; *zwf*, glucose-6-phosphate dehydrogenase coding gene; *pgl*, 6-phosphogluconolactonase coding gene; *edd*, phosphogluconate dehydratase coding gene; *eda*, KDPG aldolase coding gene; *pgi*, phosphoglucose isomerase coding gene; *gnd*, 6-phosphogluconate dehydrogenase coding gene; *tal*, transaldolase coding gene; *tkt*, transketolase coding gene; *xylB*, xylulokinase coding gene; *xylA*, xylose isomerase coding gene. Abbreviations for metabolites: G6P, glucose-6-phosphate; 6PG, 6-phosphogluconate; KDPG, 2-keto-3-deoxy-6-phosphogluconate; F6P, fructose-6-phosphate; FBP, fructose-1,6-bisphosphate; DHAP, dihydroxyacetone phosphate; G3P, glyceraldehyde-3-phosphate; E4P, erythrose-4-phosphate; S7P, sedoheptulose-7-phosphate; X5P, xylulose-5-phosphate; R5P, ribose-5-phosphate; Ru5P, ribulose-5-phosphate; EMP pathway, Embden–Meyerhof–Parnas pathway; PPP pathway, pentose phosphate pathway; ED pathway, Entner–Doudoroff pathway. (**b**) Strategy for the adaptive laboratory evolution of *E. coli* MD0 into *E. coli* MDE using M9 medium with varying mixtures of glucose, xylose, and gluconate. (**c**) Comparison of glucose and xylose consumption by *E. coli* MDEΔ*zwf* and *E. coli* MDEΔ*xylA* during shake flask fermentation with 10 g/L glucose and 10 g/L xylose as carbon sources. (**d**) Time course of glucose consumption of *E. coli* MG1655, *E. coli* MG1655Δ*ptsG*, *E. coli* MD0, and *E. coli* MDE during shake flask fermentation with 10 g/L glucose and 10 g/L xylose as carbon sources. (**e**) Time course of xylose consumption of *E. coli* MG1655, *E. coli* MG1655Δ*ptsG*, *E. coli* MD0, and *E. coli* MDE during shake flask fermentation with 10 g/L glucose and 10 g/L xylose as carbon sources. (**f**) Comparison of total sugar consumption by *E. coli* MG1655, *E. coli* MG1655Δ*ptsG*, *E. coli* MD0, and *E. coli* MDE during shake flask fermentation with 10 g/L glucose and 10 g/L xylose as carbon sources. Data shown are means ± SD (*n* = 3 independent experiments). ns, no significant difference (*P* ≥ 0.05 in two-tailed Student’s *t*-test); *P < 0.05 in two-tailed Student’s *t*-test; **P < 0.01 in two-tailed Student’s *t*-test; ***P < 0.001 in two-tailed Student’s *t*-test; ****P < 0.0001 in two-tailed Student’s *t*-test.

In addition to the EMP and PPP pathways, glucose can also be metabolized via the Entner−Doudoroff (ED) pathway in *E. coli*, albeit with extremely low carbon flux. For instance, only 2% of glucose is routed through the ED pathway in *E. coli* MG1655 (8, 9). In this study, we deleted the *pgi* and *gnd* genes in *E. coli* MG1655, generating the strain *E. coli* MG1655Δ*pgi*Δ*gnd* (MD0). Theoretically, *E. coli* MD0 can only metabolize glucose via the ED pathway, while its xylose metabolism through the EMP and PPP pathways remains unaffected (Fig. 1a). We then obtained an efficient glucose-xylose co-utilizing mutant, *E. coli* MDE, through adaptive laboratory evolution (ALE) of *E. coli* MD0 (Fig. 1b). Importantly, *E. coli* MDE with non-overlapping glucose and xylose metabolic pathways exhibits higher mixed-sugar utilization efficiency than *E. coli* MG1655Δ*ptsG*, another strain with alleviated CCR constructed by deletion of the *ptsG* gene encoding EIIBC^Glc^ in the PTS system (10–12). Furthermore, we elucidated the molecular mechanism underlying the efficient co-utilization of glucose and xylose by *E. coli* MDE. Finally, we employed *E. coli* MDE as a chassis strain for the production of value-added products, pyruvate and 2,3-butanediol (2,3-BD) from straw hydrolysate.

## RESULTS

### Obtaining mutant *E. coli* MDE with simultaneous glucose and xylose metabolism

CCR in *E. coli* is primarily realized through the PTS system, which influences cAMP synthesis and weakens the transcription activation of xylose transport and catabolic genes by cyclic AMP receptor protein (13). The *ptsG* gene encodes EIIBC^Glc^, a major component of the PTS system. Thus, interrupting PTS by knocking out *ptsG* is a commonly utilized strategy to eliminate CCR in *E. coli* (14). In this study, we proposed another strategy for the co-utilization of glucose and xylose in *E. coli*. We knocked out *pgi* and *gnd* genes in *E. coli* MG1655. The obtained strain *E. coli* MD0 can theoretically metabolize glucose through the ED pathway and utilize xylose through the EMP pathway and PPP pathway (Fig. 1a). It exhibited significant growth defects when using a mixture of glucose and xylose as the carbon source (Fig. S1), which may be due to slow metabolism of glucose with the ED pathway and inhibited xylose metabolism because of CCR.

*E. coli* MD0 can grow normally with gluconate through the ED pathway (Fig. S2) (15, 16). Thus, we conducted ALE of *E. coli* MD0 with 5 g/L glucose, 5 g/L xylose, and 2 g/L gluconate as mixed carbon sources. After 50 times sub-culturing, gluconate was eliminated from the culture medium, and the strain was sub-cultured with 5 g/L glucose and 5 g/L xylose for another 100 times. Finally, the concentrations of glucose and xylose were increased to 10 g/L, and *E. coli* MDE with efficient co-utilization of glucose and xylose was isolated after sub-culturing for 47 times (Fig. 1b). Then, we constructed *E. coli* MDEΔ*zwf* and *E. coli* MDEΔ*xylA* and cultured these two strains with 10 g/L glucose and 10 g/L xylose as mixed carbon sources. As expected, *E. coli* MDEΔ*zwf* lost the ability to metabolize glucose while maintaining normal xylose utilization, whereas *E. coli* MDEΔ*xylA* failed to consume xylose while maintaining normal glucose utilization (Fig. 1c; Fig. S3).

We compared the growth and carbon source utilization of *E. coli* MG1655, *E. coli* MD0, *E. coli* MDE, and *E. coli* MG1655Δ*ptsG*. As shown in Fig. 1d and e, *E. coli* MG1655 preferentially utilized glucose, and xylose consumption was barely detected before glucose exhaustion. *E. coli* MG1655Δ*ptsG* can simultaneously utilize glucose and xylose, but the cell growth and glucose consumption ratio partly decreased (Fig. S1). ALE of *E. coli* MD0 obviously improved the growth and utilization of both glucose and xylose. Importantly, the ratio of utilized sugars of *E. coli* MDE reached 87.7%, which was much higher than those of *E. coli* MG1655Δ*ptsG* (68.0%), *E. coli* MD0 (27.9%), and *E. coli* MG1655 (55.2%) (Fig. 1f).

### Transcriptome analysis and whole-genome sequencing of *E. coli* MDE

**Fig 2:**
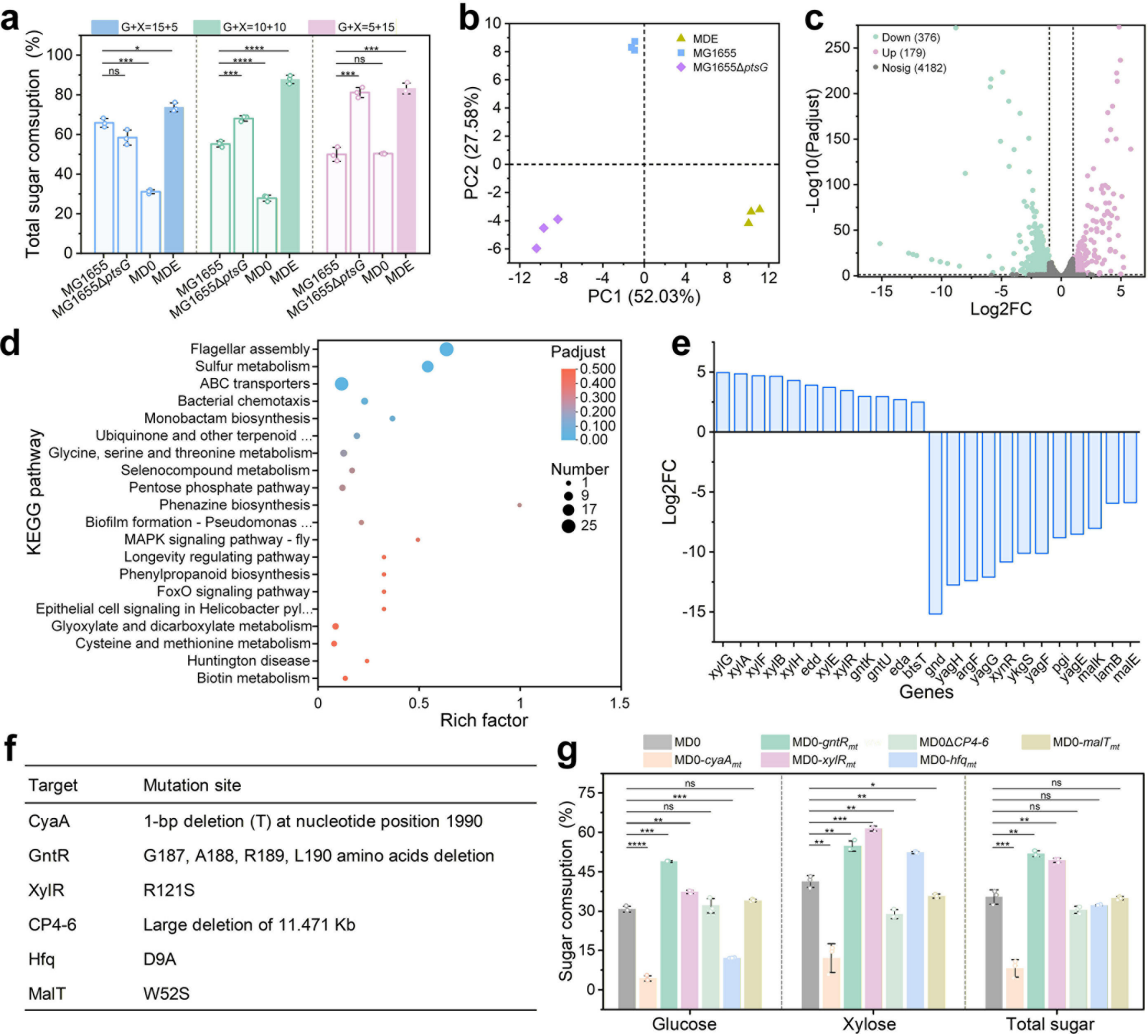
Transcriptome and whole-genome sequencing analysis of *E. coli* MDE. (**a**) Comparison of total sugar consumption by *E. coli* MG1655, *E. coli* MG1655Δ*ptsG*, *E. coli* MD0, and *E. coli* MDE during shake flask fermentation. The shake flask fermentation was carried out in a 300 mL shake flask with 15 g/L glucose + 5 g/L xylose, 10 g/L glucose + 10 g/L xylose, and 5 g/L glucose + 15 g/L xylose as carbon sources. G + X, glucose (g/L) + xylose (g/L). (**b**) Principal component analysis showing taxonomic differences among *E. coli* MDE, *E. coli* MG1655Δ*ptsG,* and *E. coli* MG1655. Strains were cultured in M9 medium supplemented with 10 g/L glucose and 10 g/L xylose as carbon sources. Three independent biological replicates were employed. PC1 (52.03%), principal component 1: 52.03% variance; PC2 (27.58%), principal component 2: 27.58% variance. (**c**) Volcano plots presenting the upregulated and downregulated genes of *E. coli* MDE compared with *E. coli* MG1655. Nosig, not significant; FC, fold change; *P*adjust, *P* value adjusted. The genes with *P*adjust <0.05 and log2FC >1 or log2FC <−1 were considered significantly changed. (**d**) Kyoto Encyclopedia of Genes and Genomes (KEGG) enrichment scatter plot of the top 20 enriched pathways (*E. coli* MDE vs *E. coli* MG1655). (**e**) Bar plot of upregulated genes (top 12) and downregulated genes (top 12) that are involved in carbon metabolism of *E. coli* MDE compared with *E. coli* MG1655. (**f**) Identified mutations in strain *E. coli* MDE. (**g**) Comparison of glucose, xylose, and total sugar consumption by *E. coli* MD0 and its derivatives during shake flask fermentation with 10 g/L glucose and 10 g/L xylose as carbon sources. Data shown are means ± SD (*n* = 3 independent experiments). ns, no significant difference (*P* ≥ 0.05 in two-tailed Student’s *t*-test); *P < 0.05 in two-tailed Student’s *t*-test; **P < 0.01 in two-tailed Student’s *t*-test; ***P < 0.001 in two-tailed Student’s *t*-test; ****P < 0.0001 in two-tailed Student’s *t*-test.

Lignocellulosic hydrolysates prepared from different biomasses exhibit significant divergence in proportions of glucose and xylose (17–19). *E. coli* MG1655, *E. coli* MD0, *E. coli* MDE, and *E. coli* MG1655Δ*ptsG* were also cultured in medium with glucose and xylose ratios of 3:1, 1:1, and 1:3 (Fig. 1d and e; Fig. S1, S4, and S5). As shown in Fig. 2a, the ratios of utilized sugars of *E. coli* MDE were higher than those of *E. coli* MG1655, *E. coli* MG1655Δ*ptsG,* and *E. coli* MD0 in different media, supporting its potential in resource utilization of different biomasses.

Then, the transcriptome analysis of strains *E. coli* MG1655, *E. coli* MG1655Δ*ptsG*, and *E. coli* MDE in media with 10 g/L glucose and 10 g/L xylose as carbon sources was performed. Principal component analysis revealed distinct differences in the transcriptional profile of *E. coli* MDE, *E. coli* MG1655, and *E. coli* MG1655Δ*ptsG* (Fig. 2b). Differential expression gene analysis identified 179 genes significantly upregulated and 376 genes significantly downregulated in *E. coli* MDE compared to *E. coli* MG1655 (Fig. 2c). Kyoto Encyclopedia of Genes and Genomes (KEGG) pathway enrichment analysis demonstrated that upregulated genes in *E. coli* MDE were mainly involved in carbon metabolism, sulfur metabolism, and flagellar assembly (Fig. 2d). The xylose utilization genes *xylAB*, *xylE*, and *xylFGHR*, the genes *edd* and *eda* involved in the ED pathway, and the gluconate transporter genes *gntKU* were significantly upregulated in *E. coli* MDE (Fig. 2e). These results demonstrate that *E. coli* MDE reconfigured its carbon metabolism by activating the xylose metabolism pathway and the ED pathway to co-utilize xylose and glucose.

Whole-genome sequencing of *E. coli* MDE was also performed. Five mutations related to *cyaA* encoding adenylate cyclase that produces the second messenger cAMP to regulate CCR (20), *xylR* encoding the xylose operon activator, *gntR* encoding the transcription factor negatively regulating the *edd-eda* operon involved in D-gluconate metabolism via the ED pathway (21, 22), *malT* encoding the maltose activator regulating the maltose regulon involved in the uptake and metabolism of maltodextrins (23), *hfq* encoding the global RNA chaperone known to control *ptsG* mRNA stability (24), were identified in *E. coli* MDE (Fig. 2f). Besides the above five mutations which may influence central carbon metabolism, a large 11.471 kb deletion, including the xylonate metabolism transcriptional repressor gene *xynR* and the xylonate metabolism gene cluster *yag* in the CP4-6 prophage region, was also identified (25). Interestingly, *xynR*, *argF*, *yagEFGH*, and the deleted genes *pgi* and *gnd* were among the top 12 downregulated genes involved in carbon metabolism of *E. coli* MDE (Fig. 2e).

Next, the five mutations and the 11.471 kb deletion were introduced into *E. coli* MD0 to compare their effect on glucose and xylose co-utilization. As shown in Fig. 2g, no obvious improvement in total sugar consumption in *E. coli* MD0ΔCP4-6, *E. coli* MD0-*hfq_mt_*, and *E. coli* MD0-*malT_mt_* was observed. *E. coli* MD0-*gntR_mt_* and *E. coli* MD0-*xylR_mt_* exhibited markedly higher ratios of total sugar consumption than that of *E. coli* MD0, while *E. coli* MD0-*cyaA_mt_* exhibited a decreased ratio of total sugar consumption. These results indicate that *gntR_mt_* and *xylR_mt_* are positive mutations responsible for increased mixed-sugar utilization of *E. coli* MDE.

### Destabilization of GntR_mt_ activates the ED pathway for enhanced glucose metabolism

**Fig 3:**
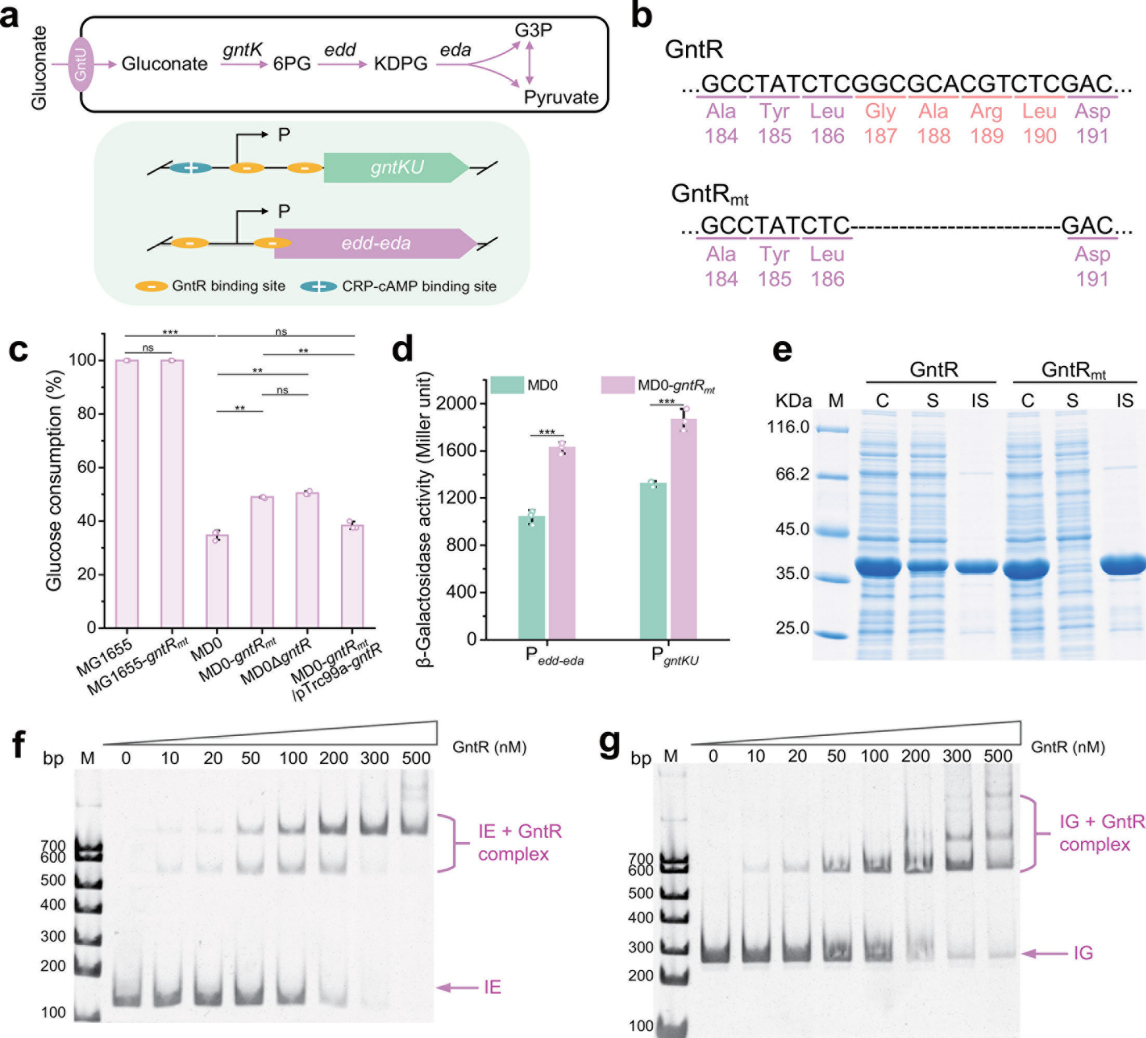
Molecular mechanism of enhanced glucose utilization in *E. coli* MDE by GntR_mt_. (**a**) Transcriptional organization of the GntR-regulated operons. GntU, low-affinity gluconate transporter; *gntK*, D-gluconate kinase encoding gene; *edd*, phosphogluconate dehydratase encoding gene; *eda*, KDPG aldolase encoding gene; 6PG, 6-phosphogluconate; KDPG, 2-keto-3-deoxy-6-phosphogluconate; G3P, glyceraldehyde-3-phosphate. (**b**) Sequence alignment of the amino acid sequences (residues 184–191) and the corresponding nucleotide sequences of GntR and GntR_mt_. (**c**) Comparison of glucose consumption by *E. coli* MG1655, *E. coli* MG1655-*gntR_mt_*, *E. coli* MD0, *E. coli* MD0-*gntR_mt_*, *E. coli* MD0Δ*gntR*, and *E. coli* MD0-*gntR_mt_*/pTrc99a-*gntR* during shake flask fermentation with 10 g/L glucose and 10 g/L xylose as carbon sources. (**d**) β-Galactosidase activity of *E. coli* MD0 and *E. coli* MD0-*gntR_mt_* containing pME6522-P*_edd-eda_* or pME6522-P*_gntKU_* at mid-log stage in M9 medium with 10 g/L glucose and 10 g/L xylose as carbon sources. (**e**) Sodium dodecyl sulfate-polyacrylamide gel electrophoresis analysis of the solubility of the GntR and GntR_mt_ proteins. Lane M, protein marker; lane C, crude extract; lane S, soluble supernatant; lane IS, insoluble sediment. (**f**) GntR can bind to the promoter region of *edd-eda*. Lane M, DNA molecular weight markers. IE, *edd-eda* promoter region. (**g**) GntR can bind to the promoter region of *gntKU*. Lane M, DNA molecular weight markers. IG, *gntKU* promoter region. Data shown are means ± SD (*n* = 3 independent experiments). ns, no significant difference (*P* ≥ 0.05 in two-tailed Student’s *t*-test); *P < 0.05 in two-tailed Student’s *t*-test; **P < 0.01 in two-tailed Student’s *t*-test; ***P < 0.001 in two-tailed Student’s *t*-test; ****P < 0.0001 in two-tailed Student’s *t*-test.

The *edd-eda* operon encodes phosphogluconate dehydratase and 2-keto-3-deoxy-gluconate 6-phosphate aldolase, the key enzymes in the ED pathway of *E. coli* (Fig. 3a) (26). The transcription of *edd* and *eda* in *E. coli* MDE was 15.0-fold and 6.5-fold higher than those in *E. coli* MG1655 (Table S1). GntR negatively regulates the expression of *edd-eda* through binding to the promoter region of *edd-eda*. The gene *gntR_mt_* in *E. coli* MDE encodes a mutated GntR (referred to as GntR_mt_) with deletion of four amino acids (Gly 187, Ala 188, Arg 189, Leu 190) (Fig. 3b).

The four amino acid deletion was introduced in GntR of *E. coli* MG1655 and *E. coli* MD0. *E. coli* MG1655 primarily metabolizes glucose through the EMP pathway and the PPP pathway. There was no obvious difference in the growth and glucose consumption of *E. coli* MG1655 and *E. coli* MG1655-*gntR_mt_* during growth with glucose as the sole carbon source (Fig. S6 ). *E. coli* MD0 can only metabolize glucose through the ED pathway . As expected, the utilization of glucose by *E. coli* MD0-*gntR_mt_* was obviously improved after mutation of *gntR* (Fig. 3c; Fig. S6). GntR also negatively regulates the expression of *gntKU*, which encodes gluconate kinase and gluconate transporter, the key proteins for gluconate transport and phosphorylation in *E. coli* (Fig. 3a) (27, 28). The promoter activities of P*_edd-eda_* and P*_gntKU_* in *E. coli* MD0 and *E. coli* MD0-*gntR_mt_* were measured by β-galactosidase assays. The results showed that the activities of promoters P*_edd-eda_* and P*_gntKU_* in *E. coli* MD0-*gntR_mt_* were significantly higher than those of *E. coli* MD0 (Fig. 3d), indicating that GntR_mt_ has positive effects on glucose metabolism through the ED pathway. We overexpressed and purified GntR of *E. coli* MG1655 (Fig. 3e). As expected, GntR binds to the promoter regions of *edd-eda* and *gntKU* (Fig. 3f and g). However, the overexpressed GntR_mt_ existed in the form of inclusion bodies, and no purified GntR_mt_ was obtained (Fig. 3e). The insolubility of the expressed GntR_mt_ may have resulted in the loss of the ability to bind with the promoter region of *edd-eda*, while the increased expression of *edd-eda* improves glucose metabolism via the ED pathway. We constructed *E. coli* MD0Δ*gntR* and overexpressed *gntR* in *E. coli* MD0-*gntR_mt_* using plasmid pTrc99a. As expected, the glucose consumption ratio of *E. coli* MD0Δ*gntR* showed no significant difference from that of *E. coli* MD0-*gntR_mt_*, while the glucose consumption ratio of *E. coli* MD0-*gntR_mt_*/pTrc99a-*gntR* was reduced to the level of *E. coli* MD0 (Fig. 3c; Fig. S6). These results indicate that *gntR_mt_* behaves simply as a loss of function.

### DNA-binding of XylR_mt_ drives efficient xylose metabolism under low xylose concentrations

**Fig 4:**
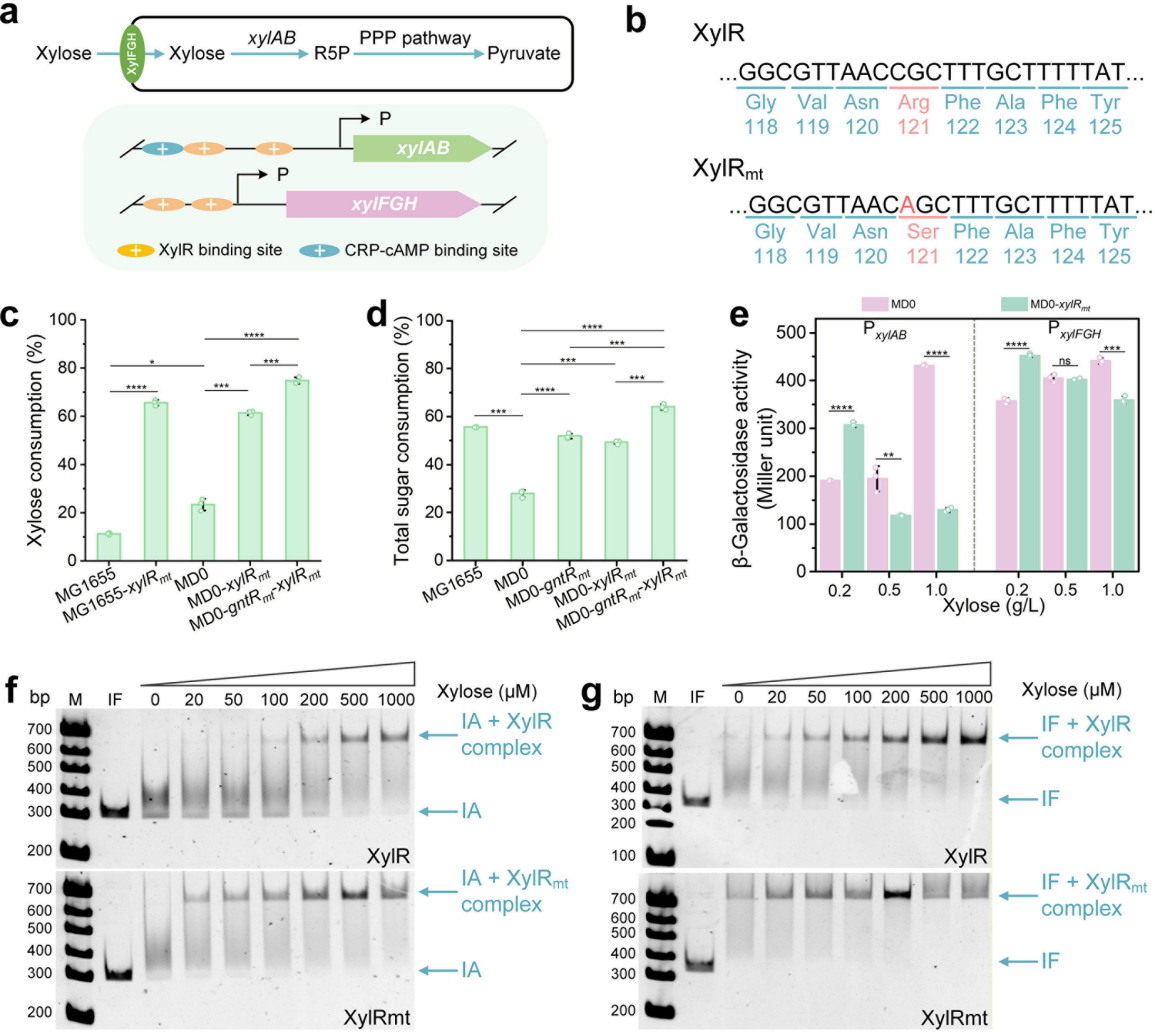
Molecular mechanism of enhanced xylose utilization in *E. coli* MDE by XylR_mt_. (**a**) Transcriptional organization of the XylR-regulated operons. XylFGH, xylose ABC transporters; X5P, xylulose-5-phosphate; *xylA*, xylose isomerase coding gene; *xylB*, xylulokinase coding gene. (**b**) Sequence alignment of the nucleotide and amino acid sequences (residues 118–125) of XylR and XylR_mt_. (**c**) Comparison of xylose consumption by *E. coli* MG1655, MG1655-*xylR_mt_*, *E. coli* MD0, *E. coli* MD0-*xylR_mt_*, and *E. coli* MD0-*gntR_mt_-xylR_mt_* during shake flask fermentation with 10 g/L glucose and 10 g/L xylose as carbon sources. (**d**) Comparison of total sugar consumption by *E. coli* MG1655, *E. coli* MD0, *E. coli* MD0-*gntR_mt_*, *E. coli* MD0-*xylR_mt_*, and *E. coli* MD0-*gntR_mt_-xylR_mt_* during shake flask fermentation with 10 g/L glucose and 10 g/L xylose as carbon sources. (**e**) β-Galactosidase activity of *E. coli* MD0 and *E. coli* MD0-*xylR_mt_* containing pME6522-P*_xylAB_* or pME6522-P*_xylFGH_* at mid-log stage in M9 medium with varying concentrations of xylose (0.2, 0.5, or 1.0 g/L) as the sole carbon source. (**f**) Effects of a gradient concentration of xylose (0–1,000 μM) on the binding ability of XylR or XylR_mt_ to *xylAB* promoter. IA, *xylAB* promoter region. (**g**) Effects of a gradient concentration of xylose (0–1,000 μM) on the binding ability of XylR or XylR_mt_ to *xylFGH* promoter. IF, *xylFGH* promoter region. Data shown are means ± SD (*n* = 3 independent experiments). ns, no significant difference (*P* ≥ 0.05 in two-tailed Student’s *t*-test); *P < 0.05 in two-tailed Student’s *t*-test; **P < 0.01 in two-tailed Student’s *t*-test; ***P < 0.001 in two-tailed Student’s *t*-test; ****P < 0.0001 in two-tailed Student’s *t*-test.

Xylose is first catalyzed by XylA to generate xylulose, and xylulose is catalyzed by XylB to generate xylulose-5-phosphate, which enters the PPP pathway for further metabolism in *E. coli* (29, 30). The transcription of *xylA* and *xylB* in *E. coli* MDE was 28.9-fold and 25.0-fold higher than those in *E. coli* MG1655 (Table S1). XylR is a xylose-specific transcription-activating protein that activates the expression of *xylAB* by binding to the promoters of *xylAB* (Fig. 4a) (31). Whole-genome sequencing indicated that a point mutation results in an amino acid change of XylR (R121S) in *E. coli* MDE (Fig. 4b).

*E. coli* MG1655-*xylR_mt_* and *E. coli* MD0-*xylR_mt_* were constructed by introducing the point mutation in *xylR*. As shown in Fig. 4c, the utilization of xylose in *E. coli* MG1655-*xylR_mt_* and *E. coli* MD0-*xylR_mt_* obviously increased (Fig. S7), indicating the positive effect of *xylR* mutation on xylose utilization. Then, the *xylR_mt_* was introduced in *E. coli* MD0-*gntR_mt_*. As expected, the ratio of utilized xylose also increased 1.9-fold in *E. coli* MD0-*gntR_mt_-xylR_mt_*. Moreover, the ratio of utilized sugars of *E. coli* MD0-*gntR_mt_-xylR_mt_* reached 64.1%, which was much higher than those of *E. coli* MD0 (27.9%) and *E. coli* MD0-*gntR_mt_* (51.8%), *E. coli* MD0-*xylR_mt_* (49.3%) with 10 g/L glucose and 10 g/L xylose as carbon sources (Fig. 4d).

XylR also activates the expression of *xylFGH*, which encodes the xylose-specific ATP-binding cassette ABC transporter in *E. coli* (Fig. 4a) (31). Transcriptome analysis demonstrated substantial upregulation of the transporter genes *xylF*, *xylG*, and *xylH* in *E. coli* MDE, with fold increases of 25.8, 31.0, and 19.7, respectively (Table S1). We quantified the activities of the promoters P*_xylAB_* and P*_xylFGH_* using β-galactosidase assays. The promoter activities of P*_xylAB_* and P*_xylFGH_* in *E. coli* MD0-*xylR_mt_* were significantly higher than those in *E. coli* MD0 under low xylose concentrations (Fig. 4e). We also overexpressed and purified XylR and XylR_mt_ (Fig. S8). The XylR_mt_ and XylR required 20 and 100 μM xylose to bind the P*_xylAB_* fragment (IA), respectively. Moreover, XylR_mt_ can bind the P*_xylFGH_* fragment (IF) even without xylose (Fig. 4f and g). These results suggest that XylR_mt_ exhibits increased DNA-binding under low xylose concentrations, which contributes to more efficient xylose utilization.

### Introducing *gntR_mt_* and *xylR_mt_* into *K. oxytoca* improves glucose and xylose co-utilization

**Fig 5:**
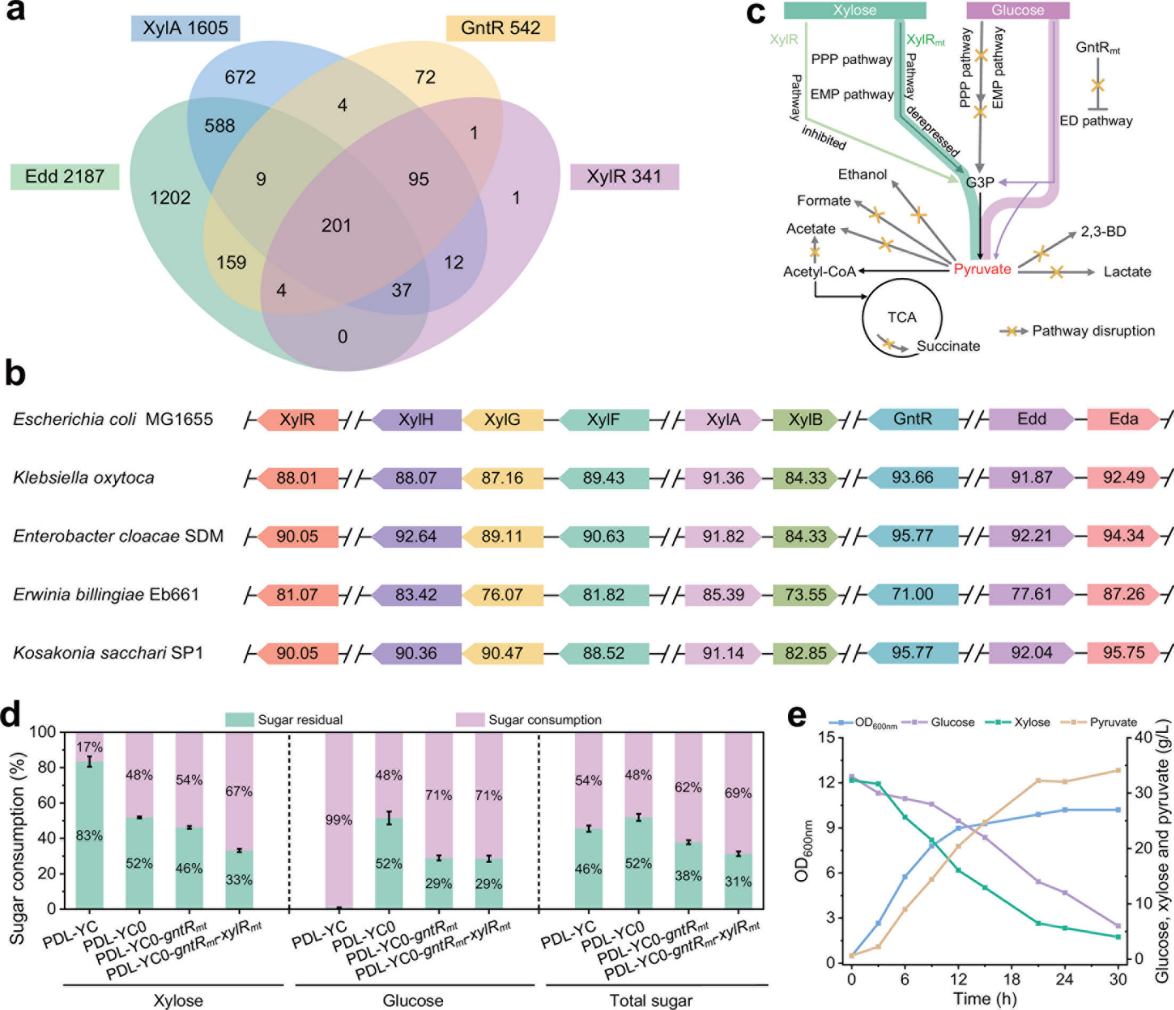
The application of the pathway separation strategy in converting sugar mixtures into chemicals. (**a**) The Venn diagram illustrates the occurrence and overlap of EDD, XylA, GntR, and XylR through context analysis of 33,478 bacterial genomes. Homologs were identified with the restriction of a maximum identity level higher than 50%, query coverage of more than 90%, and an E value lower than e^−30^. (**b**) Comparative analysis of proteins involved in the ED pathway and xylose metabolism in different bacteria. The numbers in the arrows show amino acid sequence identity (%) between *E. coli* MG1655 and other strains. XylR, xylose operon activator; XylF, xylose ABC transporter periplasmic binding protein; XylG, xylose ABC transporter ATP binding subunit; XylH, xylose ABC transporter membrane subunit; XylA, xylose isomerase; XylB, xylulokinase; GntR, gluconate transcriptional repressor; Edd, phosphogluconate dehydratase; Eda, KDPG aldolase; KDPG, 2-keto-3-deoxy-6-phosphogluconate. (**c**) Metabolic engineering strategies for pyruvate production from glucose and xylose by *K. oxytoca* PDL-YC. XylR, xylose operon activator; GntR, transcription factor. Abbreviations for metabolites: G3P, glyceraldehyde-3-phosphate; 2,3-BD, 2,3-butanediol; TCA, tricarboxylic acid cycle. (**d**) Comparison of glucose, xylose, and total sugar consumption by *K. oxytoca* PDL-YC and its derivatives during shake flask fermentation with 20 g/L glucose and 20 g/L xylose as carbon sources. Data shown are means ± SD (*n* = 3 independent experiments). (**e**) Batch fermentation using glucose and xylose as carbon sources by *K. oxytoca* PDL-YC0-*gntR_mt_-xylR_mt_* in a 1-L bioreactor. The experiment was conducted in triplicate. A representative time-course is reported herein.

XylA and Edd are the key enzymes for xylose and glucose metabolism of *E. coli* MDE, respectively. The distribution of XylA, Edd, GntR, and XylR in bacteria was studied by using the Protein BLAST program in the sequenced bacterial genomes from GenBank (GenBank, updated October 2025). Homologs were identified with the restriction of a maximum identity level higher than 50%, query coverage of more than 90%, and an E value lower than e^−30^. The homologs of XylA and Edd were found in 1,605 and 2,187 bacterial species, respectively. The homologs of regulators XylR and GntR were present in 341 and 542 species (Fig. 5a). XylA, Edd, XylR, and GntR were co-identified in approximately 201 species, including industrially relevant microorganisms, such as *Klebsiella oxytoca*, *Enterobacter cloacae*, and *Kosakonia sacchari* (Fig. 5a and b).

*K. oxytoca* PDL-YC, which exhibits strong CCR, is a pyruvate producer constructed in our previous work (32). The genes *pgi* and *gnd* were knocked out to construct *K. oxytoca* PDL-YC0 (Fig. 5c). Then, the mutant genes *gntR_mt_* and *xylR_mt_* were introduced into *K. oxytoca* PDL-YC0 for improved xylose and glucose utilization (Fig. 5d). *K. oxytoca* PDL-YC, *K. oxytoca* PDL-YC0, *K. oxytoca* PDL-YC0-*gntR_mt_*, and *K. oxytoca* PDL-YC0-*gntR_mt_-xylR_mt_* were cultured in fermentation medium supplemented with 20 g/L glucose and 20 g/L xylose as carbon sources. Due to the CCR, *K. oxytoca* PDL-YC consumed 99% of glucose and only 12% of xylose in a shake flask within 36 h (Fig. S9a). *K. oxytoca* PDL-YC0-*gntR_mt_-xylR_mt_* simultaneously consumed 71% of glucose and 67% of xylose under identical conditions (Fig. S9b). Then, batch fermentation using *K. oxytoca* PDL-YC and *K. oxytoca* PDL-YC0-*gntR_mt_-xylR_mt_* with 30 g/L glucose and 30 g/L xylose as the carbon source was conducted in a 1-L bioreactor. *K. oxytoca* PDL-YC0-*gntR_mt_-xylR_mt_* completely consumed 27.0 g/L glucose and 28.3 g/L xylose, and accumulated 34.1 g/L pyruvate in 30 h (Fig. 5e). Due to incomplete xylose consumption, *K. oxytoca* PDL-YC only accumulated 28.7 g/L pyruvate under identical conditions (Fig. S10). Thus, the strategy for glucose and xylose co-utilization of *E. coli* MDE may also be introduced to other strains with XylA, Edd, GntR, and XylR for efficient lignocellulosic bioconversion.

### Metabolic engineering of *E. coli* MDE for pyruvate and 2,3-BD overproduction from straw hydrolysate

**Fig 6:**
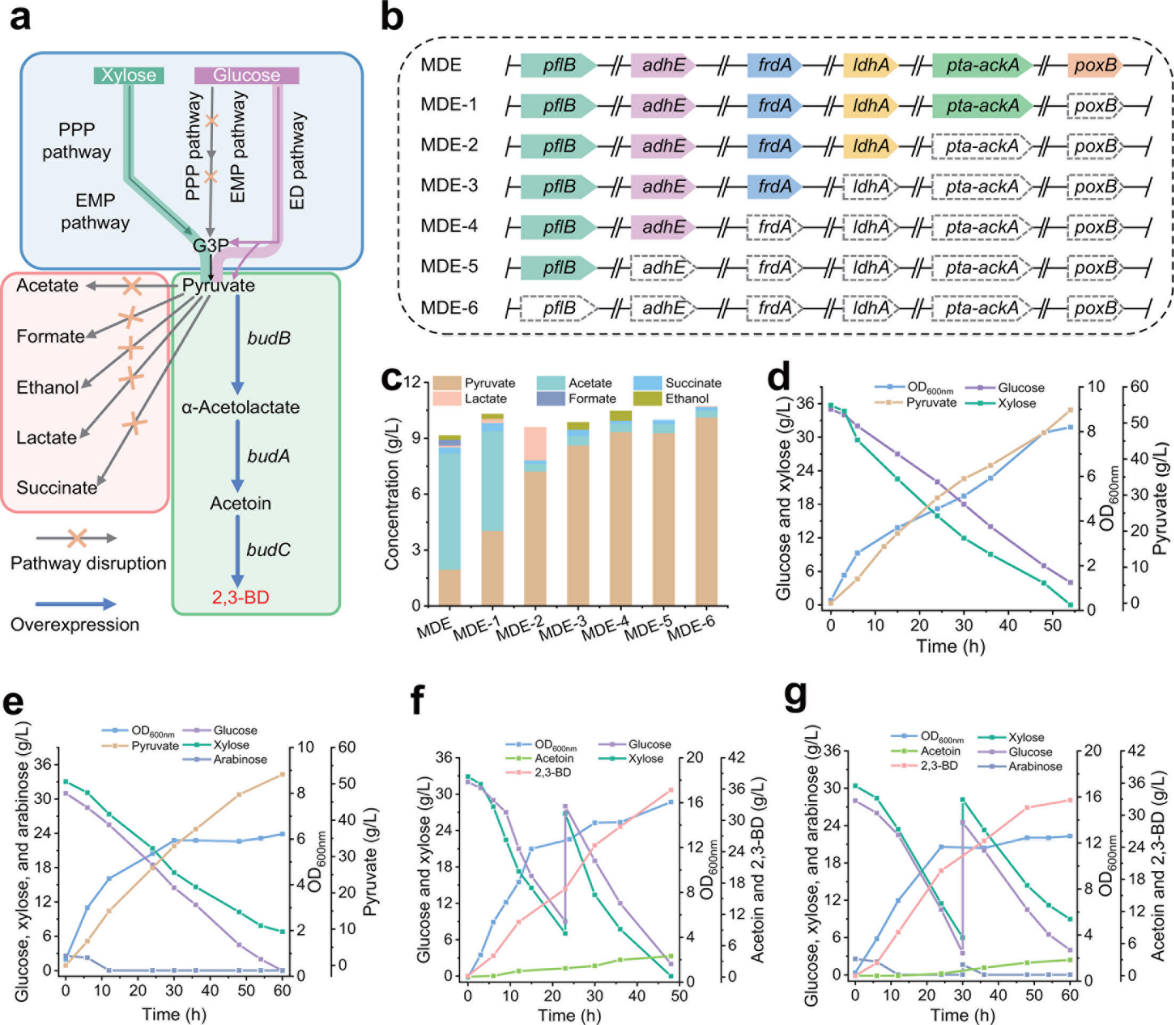
Pyruvate and 2,3-BD production from straw hydrolysate by *E. coli* MDE and its derivatives. (**a**) Metabolic engineering strategies for pyruvate and 2,3-BD production from glucose and xylose by *E. coli* MDE. *budB*, α-acetolactate synthase encoding gene; *budA*, α-acetolactate decarboxylase encoding gene; *budC*, 2,3-butanediol dehydrogenase encoding gene. Abbreviations for metabolites: G3P, glyceraldehyde-3-phosphate; 2,3-BD, 2,3-butanediol; PPP pathway, pentose phosphate pathway; ED pathway, Entner–Doudoroff pathway. (**b**) *E. coli* MDE and its derivative strains in this study. *pflB*, pyruvate formate-lyase encoding gene; *adhE*, ethanol dehydrogenase encoding gene; *frdA*, catalytic subunit of fumarate reductase encoding gene; *ldhA*, lactate dehydrogenase encoding gene; *pta*, phosphate acetyltransferase encoding gene; *ackA*, acetate kinase encoding gene; *poxB*, pyruvate oxidase encoding gene. (**c**) The concentrations of accumulated metabolites in the fermentation broth by *E. coli* MDE and its derivative strains. (**d**) Batch fermentation using glucose and xylose as carbon sources by *E. coli* MDE-6 in a 1-L bioreactor. (**e**) Batch fermentation using straw hydrolysate as a carbon source by *E. coli* MDE-6 in a 1-L bioreactor. (**f**) Fed-batch fermentation using glucose and xylose as carbon sources by *E. coli* MDE-6/pETRABC in a 1-L bioreactor. (**g**) Fed-batch fermentation using straw hydrolysate as a carbon source by *E. coli* MDE-6/pETRABC in a 1-L bioreactor. The batch and fed-batch fermentation experiments were conducted in triplicate. A representative time-course is reported herein.

Pyruvate is a key precursor for numerous important biochemicals (33–35). We first used pyruvate as the target product to assess the potential of *E. coli* MDE in chemical production. *E. coli* MDE was cultured in M9 medium supplemented with 10 g/L glucose and 10 g/L xylose as the carbon sources. As shown in Fig. S11a, *E. coli* MDE accumulated 2.0 g/L pyruvate, 6.2 g/L acetate, 0.1 g/L lactate, 0.3 g/L succinate, 0.2 g/L ethanol, and 0.3 g/L formate. The generation of these fermentation products is due to the overflow metabolism of *E. coli* under aerobic conditions (36, 37). The yield of pyruvate was only 0.1 g/g sugar. Thus, we sequentially deleted the genes *poxB*, *pta-ackA*, *ldhA*, *frdA*, *adhE*, and *pflB* in *E. coli* MDE (Fig. 6a), generating *E. coli* MDE-1, MDE-2, MDE-3, MDE-4, MDE-5, and MDE-6 (Fig. 6b). *E. coli* MDE and six recombinant strains were cultured in M9 medium with 10 g/L glucose and 10 g/L xylose as carbon sources. As expected, the deletion of these genes decreased the accumulation of by-products (Fig. 6c). The final strain *E. coli* MDE-6 consumed 7.0 g/L glucose and 11.0 g/L xylose, and produced 10.1 g/L pyruvate (Fig. S11b). The batch fermentation of *E. coli* MDE-6 was also conducted in a 1-L bioreactor. As shown in Fig. 6d, *E. coli* MDE-6 consumed 31.0 g/L glucose and 35.7 g/L xylose and produced 53.6 g/L pyruvate in 54 h. Production of pyruvate from straw hydrolysate was also investigated. As shown in Fig. 6e, *E. coli* MDE-6 could simultaneously consume both glucose and xylose in straw hydrolysate. Pyruvate at a concentration of 52.6 g/L was produced from 31.0 g/L glucose, 26.2 g/L xylose, and 2.6 g/L arabinose, with a yield of 0.88 g/g sugars.

2,3-BD is an important platform chemical with diverse applications. It can be produced from pyruvate by α-acetolactate synthase, α-acetolactate decarboxylase, and 2,3-BD dehydrogenase (38). The plasmid pETRABC carrying the 2,3-BD biosynthesis gene cluster *budRABC* from *E. cloacae* was introduced into *E. coli* MDE-6 for the production of 2,3-BD (39). Fed-batch fermentation using *E. coli* MDE-6/pETRABC with glucose and xylose as the substrates was also performed in a 1-L bioreactor. *E. coli* MDE-6/pETRABC simultaneously consumed 49.0 g/L glucose and 52.7 g/L xylose, and produced 35.8 g/L 2,3-BD and 4.0 g/L acetoin within 48 h with no detectable organic acid by-products (Fig. 6f). The yield of diol (2,3-BD plus acetoin) was 0.39 g/g sugars. Fed-batch fermentation using *E. coli* MDE-6/pETRABC with straw hydrolysate as the substrate was also performed. As shown in Fig. 6g, *E. coli* MDE-6/pETRABC consumed 45.0 g/L glucose, 43.6 g/L xylose, and 4.3 g/L arabinose and produced 32.8 g/L 2,3-BD and 3.0 g/L acetoin in 60 h. The yield of diol was 0.38 g/g sugars.

## DISCUSSION

Relieving CCR to enable efficient co-utilization of glucose and xylose is crucial for enhancing chemical production from lignocellulosic hydrolysates. In *E. coli*, CCR is primarily mediated by global transcriptional regulation and inducer exclusion triggered by the PTS. As such, researchers have commonly attempted to alleviate CCR by disrupting the PTS pathway. However, inactivation of the PTS system impairs glucose utilization (40), necessitating the overexpression of galactose permease (GalP) to restore glucose uptake (41). Alternative strategies, including constitutive expression of xylose metabolism genes or introduction of mutated *xylR*, have also been employed to facilitate glucose-xylose co-utilization in *E. coli* (42–45). Given the high overlap between glucose and xylose metabolic pathways, strategies targeting the transcriptional regulation of these pathways have not significantly improved total sugar utilization in *E. coli*.

Direct modification of glucose and xylose metabolic pathways has also been explored for glucose-xylose co-utilization in *E. coli*. For example, Gawand et al. constructed the recombinant strain *E. coli* LMSE2 by deleting *pgi*, *rpe*, and *eda* in *E. coli* MG1655 (46). *E. coli* LMSE2 can only use glucose and xylose for the synthesis of ribose-5-phosphate and xylulose-5-phosphate, respectively, and thus requires co-utilization of both sugars to generate metabolites in the PPP pathway and sustain growth. However, *E. coli* LMSE2 consumed glucose and xylose at a lower rate than the parental *E. coli* MG1655. Zhao et al. designed a “Y-shaped” microbial consortium consisting of *E. coli* EB243 and *E. coli* EB243TAM-X, two recombinant strains with distinct glucose and xylose assimilation capabilities (47). In contrast to the parental *E. coli* EB243, the microbial consortium achieved glucose-xylose co-utilization. Nevertheless, an optimal inoculum ratio of *E. coli* EB243 to *E. coli* EB243TAM-X was required to enable efficient utilization of lignocellulosic hydrolysates with specific glucose-xylose ratios. Here, we employed a pathway separation strategy combined with ALE to obtain the mutant strain *E. coli* MDE. This strain utilized 87.8% of the total sugars in media with a glucose:xylose ratio of 1:1 (Fig. 1f). Importantly, *E. coli* MDE also efficiently co-utilized glucose and xylose across media with other carbon source ratios, rendering it a robust candidate for the valorization of diverse lignocellulosic hydrolysates.

*E. coli* MDE metabolizes glucose via the ED pathway, which produces less ATP per glucose than the EMP pathway. However, the ED pathway is less thermodynamically constrained than the EMP pathway and requires less enzymatic protein to sustain the same glycolytic flux. The lower protein cost of the ED pathway can partially offset its reduced ATP yield (48). The Zwf-catalyzed reaction in the ED pathway generates NADPH. Catabolism of G3P, which is generated from glucose via the ED pathway or xylose via the PPP pathway, can support NADH production. *E. coli* MDE theoretically needs to co-utilize glucose and xylose to support its energy supply and cofactor availability. Expression of the ED pathway operon requires the presence of gluconate (49), which is negatively regulated by GntR (21). The transcription factor XylR activates the xylose metabolism genes *xylAB* and the high-affinity xylose transport genes *xylFGH* upon binding to xylose (31). In this study, we confirmed that two mutations, *gntR* and *xylR,* play pivotal roles in the efficient glucose-xylose co-utilization by *E. coli* MDE. Specifically, the deletion of four amino acids in GntR abrogated its ability to bind to the *edd-eda* promoter region, leading to gluconate-independent expression of the ED pathway operon for glucose metabolism. A point mutation in XylR (R121S) enhanced its DNA-binding capacity under low xylose concentrations, thereby improving xylose metabolism in *E. coli* MDE. We also applied the pathway separation strategy to *K. oxytoca* PDL-YC, achieving efficient glucose-xylose co-utilization in this strain (Fig. 5e). The key enzymes and regulators of the ED pathway and xylose metabolism are conserved across many bacterial strains (Fig. 5a), suggesting that this strategy could be extended to other hosts to enable efficient glucose-xylose co-utilization.

Pyruvate is a key precursor for numerous important biochemicals (33–35). In this study, *E. coli* MDE was metabolically engineered for pyruvate production, yielding the strain *E. coli* MDE-6. This strain efficiently co-utilized glucose and xylose from straw hydrolysate to produce pyruvate. Subsequently, the 2,3-BD biosynthetic pathway was further introduced into *E. coli* MDE-6 for the biosynthesis of 2,3-BD, another important platform chemical. The consumption rates of glucose and xylose during pyruvate and 2,3-BD production were higher than reported works for chemical generation from glucose and xylose with other co-utilization strategies (Table S2). Other pyruvate-derived compounds, such as l-valine (50), l-leucine (51), and 2,4-dihydroxybutyrate (52), could also be produced from straw hydrolysate through metabolic engineering of *E. coli* MDE-6. Currently, hydrolysates with varying glucose-xylose ratios have been prepared from diverse biomass feedstocks (53). Given *E. coli* MDE’s excellent performance in utilizing sugar mixtures with different glucose-xylose ratios, it also represents a promising candidate for the conversion of other lignocellulosic biomass types.

In summary, we obtained the efficient glucose-xylose co-utilizing strain *E. coli* MDE by deleting *pgi* and *gnd* in *E. coli* MG1655, followed by ALE. We further identified that mutations in *gntR* and *xylR* are responsible for the efficient glucose-xylose co-utilization by *E. coli* MDE. Additionally, we metabolically engineered *E. coli* MDE to produce the platform chemicals pyruvate and 2,3-BD from straw hydrolysate. This work provides a promising strategy for glucose-xylose co-utilization in other organisms. *E. coli* MDE and its derivatives, such as *E. coli* MDE-6, hold great potential for application in the production of various chemicals from diverse lignocellulosic biomasses.

## MATERIALS AND METHODS

### Chemicals

Xylose (99%) was purchased from Shandong Xitang Biotech Co., Ltd. (Jinan, China). 2,3-BD and acetoin were purchased from Sigma (USA). Straw hydrolysate (Q/AQJM 2595-2025) containing 252 g/L glucose, 272.78 g/L xylose, and 25.48 g/L arabinose was obtained from Angel Yeast Co., Ltd. (Hubei, China). All other chemicals are commercially available.

### Bacterial strains, plasmids, and culture conditions

The strains and plasmids used in this study are listed in Tables S3 and S4. The *E. coli* MDE has undergone a patent deposit in the China Center for Type Culture Collection (CCTCC) under the accession number CCTCC M 2026821. *E. coli* and *K. oxytoca* were generally cultivated in lysogeny broth (LB) medium at 37°C and 180 rpm. Plasmids pEcgRNA and pEcCas were employed for gene knockout or replacement in *E. coli*. Plasmids pEcgRNA and pEcCas_cm_ were employed for gene knockout or replacement in *K. oxytoca*. Antibiotics, including kanamycin, chloramphenicol, and spectinomycin, were used at the concentrations of 50, 40, and 50 μg/mL, respectively.

### Gene knockout and replacement

The primers used in this study are listed in Table S5. The recombinant *E. coli* MG1655 and *K. oxytoca* were constructed through the CRISPR-Cas9 system as described previously (54). The knockout of *pgi* in *E. coli* MG1655 was taken as an example. The primer pair gRNA-*pgi*-F/gRNA-*pgi*-R was annealed to form dsDNA targeting *pgi*. Then, the dsDNA was ligated into linearized pEcgRNA using T4 DNA ligase, resulting in plasmid pEcgRNA-Δ*pgi*. To obtain donor DNA, the upstream and downstream homologous arms of *pgi* were amplified using primer pairs Δ*pgi*-F1/Δ*pgi*-R1 and Δ*pgi*-F2/Δ*pgi*-R2 from the genome of *E. coli* MG1655. These two fragments were fused via PCR using primers Δ*pgi*-F1 and Δ*pgi*-R2. Then, the donor DNA and the plasmid pEcgRNA-Δ*pgi* were co-transformed into *E. coli* MG1655 harboring the plasmid pEcCas by electroporation. The correct colonies were screened out from LB plates containing 50 µg/mL spectinomycin and 50 µg/mL kanamycin, and then cultured in LB supplemented with 10 mM rhamnose and 10% (wt/vol) sucrose to induce the curing of the pEcCas and pEcgRNA-Δ*pgi* plasmids. Other *E. coli* MG1655 mutants were obtained using the same approach as described above. The *K. oxytoca* mutants were obtained using the same approach as described above, except that the plasmid pEcCas was replaced by pEcCas_cm_. pEcCas_cm_ is a modified form of pEcCas that confers chloramphenicol resistance.

### Adaptive laboratory evolution

ALE was performed on *E. coli* MD0. In the first stage, *E. coli* MD0 was inoculated (1%, vol/vol) into shake flasks containing 50 mL of M9 medium supplemented with 5 g/L glucose, 5 g/L xylose, and 2 g/L gluconate, and cultured at 37°C and 180 rpm. The culture was then transferred into fresh medium (1%, vol/vol) for subsequent cultivation and passaged 50 times. In the second stage, the carbon sources were adjusted to 5 g/L glucose and 5 g/L xylose, and the culture was passaged 100 times. In the third stage, the carbon sources were changed to 10 g/L glucose and 10 g/L xylose, and the culture was passaged 47 times. A single colony was isolated by plate streaking to obtain the evolved strain, named *E. coli* MDE.

### Transcriptome analysis

*E. coli* MG1655, *E. coli* MG1655Δ*ptsG,* and *E. coli* MDE were cultured to an OD_600nm_ value of 1 in M9 medium containing 10 g/L glucose and 10 g/L xylose as carbon sources. Cells were quick-frozen in liquid nitrogen. Transcriptional profiling was performed by Majorbio Bio-Pharm Technology Co., Ltd. (Shanghai, China). Total RNA was extracted using TRIzol Reagents (Invitrogen, USA). Sequencing library was generated from targeted RNA using the TruSeq RNA sample preparation Kit (Illumina, CA). The libraries were sequenced using an Illumina NovaSeq 6000. The clean reads were aligned to the *E. coli* MG1655 reference genome using Bowtie2, RSeQC software package. The differential expression analysis was conducted using DESeq2. Transcriptomic analysis was performed using Majorbio Cloud.

### Genomic DNA resequencing of *E. coli* MDE

Genomic DNA of *E. coli* MDE was extracted using the Wizard Genomic DNA purification kit according to the manufacturer’s protocol. The concentration of purified genomic DNA was measured with Nanodrop 2500. Whole-genome sequencing was performed by Majorbio Bio-Pharm Technology Co., Ltd. (Shanghai, China) using the Illumina HiSeq and the PacBio RSII system. Then, the clean short reads and HiFi reads were assembled to construct complete genomes using SOAPdenovo v2.04 and GapCloser v1.12. The complete genome was aligned against the reference genome *E. coli* MG1655.

### Batch fermentation in shake flask

*E. coli* MG1655 and its derivatives were cultured in M9 medium (55) with different carbon sources at 180 rpm and 37°C. *K. oxytoca* and its derivatives were cultured in fermentation medium (32) with 20 g/L glucose and 20 g/L xylose at 180 rpm and 37°C. During the fermentation, a 5 M NaOH solution was added to maintain a pH value of 7.4. Samples were taken every 6 h for analysis of biomass, glucose consumption, and xylose consumption.

### Fermentation in 1-L bioreactor

Batch fermentation for pyruvate production by *K. oxytoca* was conducted in a 1-L bioreactor (Infors AG, Switzerland) with 0.8 L medium. The seed culture was inoculated (10%, vol/vol) into the fermentation medium supplemented with 5 g/L yeast extract, 30 g/L glucose, and 30 g/L xylose. The cultivation was carried out at 37°C, airflow at 1.6 vvm, and stirring at 500 rpm. The initial pH was set to 7.0 and maintained by automatic addition of 10 M NaOH.

Batch fermentation for pyruvate production by *E. coli* was conducted in a 1-L bioreactor with 0.8 L medium. The seed culture was inoculated (10%, vol/vol) into the M9 medium supplemented with 30 g/L glucose and 30 g/L xylose. The cultivation was carried out at 37°C, airflow at 1.2 vvm, and stirring at 400 rpm. The initial pH was set to 7.0 and maintained by automatic addition of 10 M NaOH. Fed-batch fermentation for 2,3-BD production by *E. coli* was conducted under the same conditions. Pyruvate and 2,3-BD production was also carried out with straw hydrolysate from Angel Yeast Co., Ltd. (Hubei, China) as the substrate. Straw hydrolysate was added to the medium to make the glucose concentration about 28 g/L and the xylose concentration about 32 g/L, and supplemented when residual glucose and xylose concentrations were reduced to about 10 g/L.

### Analytical methods

Cell density was measured at 600 nm using a visible spectrophotometer (V-5100H, China). The concentration of glucose was detected by a bioanalyzer (SBA-40D, China). The concentrations of xylose, pyruvate, and other byproducts were analyzed using an HPLC system (LC-20AT, Japan) equipped with an Aminex HPX-87H column (300 × 7.8 mm, Bio-Rad, USA). H_2_SO_4_ at a concentration of 10 mM was used as the mobile phase at a flow rate of 0.4 mL/min, and the column temperature was 55°C. The concentration of 2,3-BD and acetoin was measured by GC (GC2014C, Japan) using a capillary GC column as described previously (56).

### Expression and purification of proteins

The purification of GntR was taken as an example. The *gntR* gene was amplified from the genomic DNA of *E. coli* MG1655 using primers GntR-F/GntR-R and cloned into the vector pET28a. The recombinant plasmid pET28a-GntR was introduced into *E. coli* BL21(DE3). The constructed strain was then grown to an OD_600_ of 0.6 to 0.8 in LB medium at 37°C and 180 rpm. Then, 1 mM isopropyl-β-D-thiogalactopyranoside was added to induce protein expression at 16°C and 160 rpm for 12 h. Bacterial cultures were harvested, washed once with phosphate-buffered saline, and suspended in buffer A (20 mM sodium phosphate, 20 mM imidazole, and 20 mM NaCl, pH 7.4) containing 1 mM phenylmethanesulfonyl fluoride and 10% (vol/vol) glycerol. Then, the cells were lysed using a high-pressure homogenizer (ATS, China). The lysate was centrifuged at 12,000 × *g* for 40 min at 4°C. The supernatant was loaded onto a 5 mL HisTrap HP column (GE Healthcare, USA) equilibrated with buffer A and eluted with buffer B (20 mM sodium phosphate, 500 mM imidazole, and 20 mM NaCl, pH 7.4). The purified protein was analyzed by 13% sodium dodecyl sulfate-polyacrylamide gel electrophoresis. The protein concentration was determined using a Bradford protein assay kit (Sangon, China). XylR and XylR_mt_ were expressed and purified by using the same procedure.

### Electrophoretic mobility shift assays

The DNA fragments of the *edd-eda* promoter and *gntKU* promoter were amplified from the genomic DNA of *E. coli* MG1655 using primers P*_edd-eda_*-F/P*_edd-ed_*-R and P*_gntKU_*-F/P*_gntKU_*-R (Table S4), respectively. The 20 μL reaction solution contained 10 nM DNA fragments and a gradient concentration of purified GntR in the binding buffer (pH 7.4, 10 mM Tris-HCl, 50 mM KCl, 0.5 mM EDTA, 10% glycerol, 1 mM dithiothreitol). The mixed solutions were incubated at 30°C for 30 min and loaded on 6% native polyacrylamide gels. Electrophoresis was performed at 170 V in 1× TBE buffer (pH 8.3) for 45 min on ice. The gels were stained with SYBR Green I (TaKaRa, China) for 20 min and then photographed using a G:BOX F3 gel documentation system (Syngene, USA).

To study the effect of xylose concentrations on the binding of XylR and XylR_mt_ to DNA fragments (P*_xylAB_* or P*_xylFGH_*), an electrophoretic mobility shift assay was conducted using the same procedure as described above. The 20 μL reaction solution contained 2.5 nM DNA fragments, 25 nM XylR or XylR_mt_, and a gradient concentration of xylose.

### β-Galactosidase activity assays

The promoter region fragments P*_edd-eda_*, P*_gntKU_*, P*_xylAB_*, and P*_xylFGH_* were ligated with pME6522 to obtain plasmids pME6522-P*_edd-eda_*, pME6522-P*_gntKU_*, pME6522-P*_xylAB_*, and pME6522-P*_xylFGH_*. pME6522-P*_edd-eda_* and pME6522-P*_gntKU_* were transferred into *E. coli* MD0 and *E. coli* MD0-*gntR_mt_*. pME6522-P*_xylAB_* and pME6522-P*_xylFGH_* were transferred into *E. coli* MD0 and *E. coli* MD0-*xylR_mt_*.

*E. coli* MD0 and *E. coli* MD0-*gntR_mt_* harboring pME6522-P*_edd-eda_* or pME6522-P*_gntKU_* were grown in M9 medium supplemented with 10 g/L glucose and 10 g/L xylose as carbon sources and harvested at the mid-log stage by centrifugation at 8,000 rpm for 5 min. *E. coli* MD0 and *E. coli* MD0-*xylR_mt_* harboring pME6522-P*_xylAB_* or pME6522-P*_xylFGH_* were grown in M9 medium supplemented with varying concentrations of xylose (0.2, 0.5, or 1.0 g/L) as the sole carbon source and harvested at the mid-log stage by centrifugation at 8,000 rpm for 5 min. The β-galactosidase activity was then determined using *o*-nitrophenyl-β-d-galactopyranoside as described previously (57).

### Statistical analysis

All shake-flask experiments were performed in triplicate. The data are presented as mean ± standard deviation (SD). Error bars in the figures represent SD. The statistical significance was analyzed by a two-tailed Student’s *t*-test. A *P* value less than 0.05 was considered significant (*P < 0.05, **P < 0.01; ***P < 0.001, ****P < 0.0001). Software for initial data processing was Microsoft Excel 2013, and subsequent analysis was carried out using Origin (OriginLab). Venn diagrams of the distribution of XylA, Edd, XylR, and GntR were drawn by the web tool in Bioinformatics and Systems Biology. Detailed data analysis is described above.

## Data Availability

Raw Illumina sequence data of strains *E. coli* MG1655, *E. coli* MG1655 Δ*ptsG*, and *E. coli* MDE are available at NCBI under Bioproject number PRJNA1397878.

## SUPPLEMENTAL MATERIAL

Supplemental material mbio.01150-26-s0001.docx Fig. S1 to S11; Tables S1 to S5.

ASM does not own the copyrights to Supplemental Material that may be linked to, or accessed through, an article. The authors have granted ASM a non-exclusive, world-wide license to publish the Supplemental Material files. Please contact the corresponding author directly for reuse.
