# Supplementary material for: Pathway remodeling and adaptive evolution enable efficient co-utilization of glucose and xylose in Escherichia coli
Source: mBio. 2026 Jul 14;17(8):e01150-26. doi: 10.1128/mbio.01150-26 (PMC13464349; doi:10.1128/mbio.01150-26)
Supplement: Supplementary file 1 [file mbio.01150-26-s0001.docx]

**Supporting Information**

**Pathway remodeling and adaptive evolution enable efficient co-utilization of glucose and xylose in *Escherichia coli***

Xiaoxu Tan^a^, Yichun Hu^a^, Kai Li^b^, Wengsi Meng^a,#^, Kaiyu Gao^a^, Qiaoyue Yang^a^, Lingru Gong^a^, Leilei Guo^a^, Mengyao Hao^a^, Hongxu Zhang^a^, Yidong Liu^a^, Zhaoqi Kang^a^, Cuiqing Ma^a,#^, Chao Gao^a^, Qianjin Kang^c^, Chuanjuan Lü^a^, Ping Xu^c,d,#^

^a^State Key Laboratory of Microbial Technology, Shandong University, Qingdao, People’s Republic of China

^b^Shanghai Changing Biotechnology Co., Ltd, Shanghai, People’s Republic of China

^c^State Key Laboratory of Microbial Metabolism, Joint International Research Laboratory of Metabolic & Developmental Sciences, and School of Life Sciences & Biotechnology, Shanghai Jiao Tong University, Shanghai, People’s Republic of China.

^d^SJTU Asia-Pacific Graduate Institute (SJTU-APGI), 1 Create Way, #14-01 CREATE Tower, Singapore 138602

^#^ Corresponding authors:

Mailing address for Wensi Meng: State Key Laboratory of Microbial Technology, Shandong University, Qingdao 266237, People’s Republic of China, Tel.: +86-532-58631559, E-mail: mengwensi123@126.com.

Mailing address for Cuiqing Ma: State Key Laboratory of Microbial Technology, Shandong University, Qingdao 266237, People’s Republic of China, Tel.: +86-532-58631559, E-mail: macq@sdu.edu.cn.

Mailing address for Ping Xu: State Key Laboratory of Microbial Metabolism, and School of Life Sciences & Biotechnology, Shanghai Jiao Tong University, Shanghai 200240, People’s Republic of China, Tel/Fax: +86-21-34206723, E-mail: pingxu@sjtu.edu.cn.

**Figure S1.** Growth of *E. coli* MG1655, *E. coli* MG1655Δ*ptsG*, *E. coli* MD0, and *E. coli* MDE during shake flask fermentation with 10 g/L glucose and 10 g/L xylose as carbon sources. Data shown are means ± SD (n = 3 independent experiments).

**Figure S2.** Growth and gluconate consumption of *E. coli* MD0 during shake flask fermentation with 10 g/L gluconate as carbon source. Data shown are means ± SD (n = 3 independent experiments).

**Figure S3.** Growth (a), glucose consumption (b), and xylose consumption (c) of *E. coli* MDEΔ*xylA* and *E. coli* MDEΔ*zwf* during shake flask fermentation with 10 g/L glucose and 10 g/L xylose as carbon sources. Data shown are means ± SD (n = 3 independent experiments).

**Figure S4.** Growth (a), glucose consumption (b), and xylose consumption (c) of *E. coli* MG1655, *E. coli* MG1655Δ*ptsG*, *E. coli* MD0 and *E. coli* MDE during shake flask fermentation with 5 g/L glucose and 15 g/L xylose as carbon sources. Data shown are means ± SD (n = 3 independent experiments).

**Figure S5.** Growth (a), glucose consumption (b), and xylose consumption (c) of *E. coli* MG1655, *E. coli* MG1655Δ*ptsG*, *E. coli* MD0 and *E. coli* MDE during shake flask fermentation with 15 g/L glucose and 5 g/L xylose as carbon sources. Data shown are means ± SD (n = 3 independent experiments).

**Figure S6.** Growth (a) and glucose consumption (b) of *E. coli* MG1655, *E. coli* MG1655-*gntR_mt_*, *E. coli* MD0, *E. coli* MD0-*gntR_mt_*, *E. coli* MD0Δ*gntR*, and *E. coli* MD0-*gntR_mt_*/pTrc99a-*gntR* during shake flask fermentation with 10 g/L glucose and 10 g/L xylose as carbon sources. Data shown are means ± SD (n = 3 independent experiments).

**Figure S7** Growth (a) and xylose consumption (b) of *E. coli* MG1655, *E. coli* MG1655-*xylR_mt_*, *E. coli* MD0, *E. coli* MD0-*xylR_mt_*, and MD0-*gntR_mt_*-*xylR_mt_* during batch fermentation with 10 g/L glucose and 10 g/L xylose as carbon sources. Data shown are means ± SD (n = 3 independent experiments).

**Figure S8.** SDS-PAGE analysis of purified XylR and XylRmt. Lane M, protein marker.

**Figure S9.** Growth, glucose consumption, xylose consumption, and pyruvate production of *K. oxytoca* PDL-YC (a) and *K. oxytoca* PDL-YC0-*gntR_mt_*-*xylR_mt_* (b) during shake flask fermentation with 20 g/L glucose and 20 g/L xylose as carbon sources. Data shown are means ± SD (n = 3 independent experiments).

**Figure S10.** Growth, glucose consumption, xylose consumption, and pyruvate production of *K. oxytoca* PDL-YC in a 1-L bioreactor. The experiment was conducted in triplicate. A representative time-course is reported herein.

**Figure S11.** Growth, glucose consumption, xylose consumption, pyruvate production, and byproducts accumulation of *E. coli* MDE (a) and *E. coli* MDE-6 (b) during shake flask fermentation with 10 g/L glucose and 10 g/L xylose as carbon sources. Data shown are means ± SD (n = 3 independent experiments).

**Table S1.** Top 12 up-regulated genes and top 12 down-regulated genes that involved in carbon metabolism of *E. coli* MDE compared to *E. coli* MG1655

| Gene | FC (MDE/MG1655) | Log2FC (MDE/MG1655) |
| --- | --- | --- |
| **Upregulated** |  |  |
| *xylG* | 31.016 | 4.954959609 |
| *xylA* | 28.905 | 4.853229013 |
| *xylF* | 25.813 | 4.690019268 |
| *xylB* | 25.045 | 4.64645639 |
| *xylH* | 19.680 | 4.298681539 |
| *edd* | 14.954 | 3.902494591 |
| *xylE* | 13.256 | 3.72859271 |
| *xylR* | 10.976 | 3.45625605 |
| *gntK* | 7.812 | 2.965626439 |
| *gntU* | 7.760 | 2.956114988 |
| *eda* | 6.523 | 2.705515576 |
| *btsT* | 5.624 | 2.491561666 |
| **Downregulated** |  |  |
| *gnd* | 0 | -15.16738322 |
| *yagH* | 0 | -12.75437045 |
| *argF* | 0 | -12.38864314 |
| *yagG* | 0 | -12.75437045 |
| *xynR* | 0.001 | -10.8329045 |
| *ykgS* | 0.001 | -10.10416364 |
| *yagF* | 0.001 | -10.11023088 |
| *pgi* | 0.002 | -8.811769092 |
| *yagE* | 0.003 | -8.514243797 |
| *malK* | 0.004 | -8.026958484 |
| *lamB* | 0.016 | -5.931716853 |
| *malE* | 0.017 | -5.879375583 |

Table S2 Summary of some representative strategies for co-utilization of glucose and xylose in *E. coli*

| Strain | Co-utilization strategy | Fermentation method | Product | Concentration (g/L) | Glucose consumption rate ^a^ (g/L/h) | Xylose  consumption rate ^a^ (g/L/h) | Reference |
| --- | --- | --- | --- | --- | --- | --- | --- |
| *E. coli* SCD78 | Deletion of *ptsG*, and ALE in AM1 mineral salt medium containing xylose | Batch fermentation in 500 mL bioreactor | Ethanol | 8.99 | 0.28 | 0.26 | 1 |
| *E. coli* GX1xMA | Deletion of *ptsHI*, *eda*, *ppc*, *pck*, *ppsA*, *pykF*, *pykA*; overexpression of *galp* and *glk* | Batch fermentation in shake flask | *cis*, *cis*-Muconic acid | 4.09 | 0.20 | 0.08 | 2 |
| *E. coli* LN23C | ALE of *E. coli* XW043 in AM1 mineral salt medium containing 100 g/L xylose to obtain XylR (XylR^R121C, P363S^), and introduced into TG114 | Batch fermentation in 500 mL bioreactor | Lactate | 86 | 0.52 | 0.46 | 3 |
| *E. coli* AF25 | Expression of XylR mutant (XylR^R121C, P363S^) | Batch fermentation in 500 mL bioreactor | Ethanol | 56 | 0.73 | 0.42 | 4 |
| *E. coli* LMSE2 | Deletion of *pgi*, *eda*, *rpe* | Batch fermentation in 5-L bioreactor | NR^b^ | NR^b^ | 0.34 | 0.34 | 5 |
| *E. coli* EB243 and *E. coli* EB243TAM-X | Co-culture of EB243 and EB243TAM-X. The EB243TAM-X was obtained by deleting *ptsG*, *glk*, and overexpression of *xylE*, *xylFGH*, *xylAB*, *rpe*, *tktA*, *rpiA*, *talB* in EB243 | Batch fermentation in 7.5-L bioreactor | Butanol | 20.82 | 0.43 | 0.39 | 6 |
| *E. coli* MDE-6 | Deletion of *pgi*, *gnd*, and ALE in M9 mineral salt medium containing glucose and xylose | Batch fermentation in 1-L bioreactor | Pyruvate | 53.60 | 0.57 | 0.66 | This study |
| *E. coli* MDE-6 /pETRABC | Deletion of *pgi*, *gnd*, and ALE in M9 mineral salt medium containing glucose and xylose | Fed-batch fermentation in 1-L bioreactor | 2,3-BD | 35.80 | 1.02 | 1.10 | This study |

^a^ The consumption rate of glucose or xylose is roughly calculated from the data in Figures of the related reference.

^b^ Not reported

**Table S3.** Strains used in this study.

| Strain | Characteristic | Reference or source |
| --- | --- | --- |
| *E. coli* DH5α | F^–^, φ80d/*lacZ*∆M15 ∆(*lacZYA-argF*) U169 *deoR rec*A1 *end*A1 *hsd*R17(rk^–^, mk^+^) *pho*A *sup*E44 λ^–^ *thi*^–1^ *gyr*A96 *rel*A1 | Novagen |
| *E. coli* BL21 (DE3) | F^–^ *ompT hsdSB* (r_B_^-^ m_B_^-^) *gal* (λ *c I* 857 *ind1 sam7 nin5* *lac*UV5-T7*gene1*) *dcm* (DE3) | Invitrogen |
| *E. coli* BL21 (DE3)/pET28a-GntR | *E. coli* BL21 (DE3) harboring the plasmid pET28a-GntR | This study |
| *E. coli* BL21 (DE3)/pET28a-XylR | *E. coli* BL21 (DE3) harboring the plasmid pET28a-XylR | This study |
| *E. coli* BL21 (DE3)/pET28a-XylR_mt_ | *E. coli* BL21 (DE3) harboring the plasmid pET28a-XylR_mt_ | This study |
| *E. coli* MG1655 | Wild-type strain | This study |
| *E. coli* MG1655Δ*ptsG* | *E. coli* MG1655 knocking out gene *ptsG* | This study |
| *E. coli* MD0 | *E. coli* MG1655Δ*pgi*Δ*gnd* | This study |
| *E. coli* MD0-*cyaA_mt_* | *E. coli* MD0 *cyaA*::*cyaA_mt_* | This study |
| *E. coli* MG1655-*gntR_mt_* | *E. coli* MG1655 *gntR*::*gntR_mt_* | This study |
| *E. coli* MD0-*gntR_mt_* | *E. coli* MD0 *gntR*::*gntR_mt_* | This study |
| *E. coli* MD0Δ*gntR* | *E. coli* MD0 knocking out gene *gntR* | This study |
| *E. coli* MD0-*gntR_mt_*/pTrc99a-*gntR* | *E. coli* MD0-*gntR_mt_* containing plasmid pTrc99a-*gntR* | This study |
| *E. coli* MG1655-*xylR_mt_* | *E. coli* MG1655 *xylR*::*xylR_mt_* | This study |
| *E. coli* MD0-*xylR_mt_* | *E. coli* MD0 *xylR*::*xylR_mt_* | This study |
| *E. coli* MD0Δ*CP4-6* | *E. coli* MD0 knocking out gene *CP4-6* | This study |
| *E. coli* MD0-*hfq_mt_* | *E. coli* MD0 *hfq*::*hfq_mt_* | This study |
| *E. coli* MD0-*malT_mt_* | *E. coli* MD0 *malT*::*malT_mt_* | This study |
| *E. coli* MD0-*gntR_mt_*-*xylR_mt_* | *E. coli* MD0*-gntR _mt_* *xylR*::*xylR_mt_* | This study |
| *E. coli* MDE | Isolated individual clone after adaptation of *E. coli* MD0 | This study |
| *E. coli* MDEΔ*zwf* | *E. coli* MDE knocking out gene *zwf* | This study |
| *E. coli* MDEΔ*xylA* | *E. coli* MDE knocking out gene *xylA* | This study |
| *E. coli* MD0/pME6522-P*_edd-eda_* | *E. coli* MD0 containing plasmid pME6522-P*_edd-eda_* | This study |
| *E. coli* MD0/pME6522-P*_gntKU_* | *E. coli* MD0 containing plasmid pME6522-P*_gntKU_* | This study |
| *E. coli* MD0/pME6522-P*_gntT_* | *E. coli* MD0 containing plasmid pME6522-P*_gntT_* | This study |
| *E. coli* MD0-*gntR_mt_*/pME6522-P*_edd-eda_* | *E. coli* MD0-*gntR_mt_* containing plasmid pME6522-P*_edd-eda_* | This study |
| *E. coli* MD0-*gntR_mt_*/pME6522-P*_gntKU_* | *E. coli* MD0-*gntR_mt_* containing plasmid pME6522-P*_gntKU_* | This study |
| *E. coli* MD0-*gntR_mt_*/pME6522-P*_gntT_* | *E. coli* MD0-*gntR_mt_* containing plasmid pME6522-P*_gntT_* | This study |
| *E. coli* MD0/pME6522-P*_xylAB_* | *E. coli* MD0 containing plasmid pME6522-P*_xylAB_* | This study |
| *E. coli* MD0/pME6522-P*_xylFGH_* | *E. coli* MD0 containing plasmid pME6522-P*_xylFGH_* | This study |
| *E. coli* MD0-*xylR_mt_*/pME6522-P*_xylAB_* | *E. coli* MD0-*xylR_mt_* containing plasmid pME6522-P*_xylAB_* | This study |
| *E. coli* MD0-*xylR_mt_*/pME6522-P*_xylFGH_* | *E. coli* MD0-*xylR_mt_* containing plasmid pME6522-P*_xylFGH_* | This study |
| *E. coli* MDE-1 | *E. coli* MDEΔ*poxB* | This study |
| *E. coli* MDE-2 | *E. coli* MDE-1Δ*pta-ackA* | This study |
| *E. coli* MDE-3 | *E. coli* MDE-2Δ*ldhA* | This study |
| *E. coli* MDE-4 | *E. coli* MDE-3Δ*frdA* | This study |
| *E. coli* MDE-5 | *E. coli* MDE-4Δ*adhE* | This study |
| *E. coli* MDE-6 | *E. coli* MDE-5Δ*pflB* | This study |
| *E. coli* MDE/pETRABC | *E. coli* MDE containing plasmid pETRABC | This study |
| *E. coli* MDE-6/pETRABC | *E. coli* MDE-6 containing plasmid pETRABC | This study |
| *K. oxytoca* PDL-YC | *K. oxytoca* Δ*budA*Δ*budB*Δ*adhE*Δ*pta*-*ackA*Δ*pox*Δ*frdA*Δ*pflB*Δ*yjiY*Δ*cstA*Δ*ldhD*::nox | Lab stock |
| *K. oxytoca* PDL-YC0 | *K. oxytoca* PDL-YCΔ*pgi*Δ*gnd* | This study |
| *K. oxytoca* PDL-YC0-*gntR_mt_* | *K. oxytoca* PDL-YC0 *gntR*::*gntR_mt_* | This study |
| *K. oxytoca* PDL-YC0-*gntR_mt_*-*xylR_mt_* | *K. oxytoca* PDL-YC0-*gntR_mt_* *xylR*::*xylR_mt_* | This study |

**Table S4.** Plasmids used in this study.

| Plasmid | Characteristic | Reference or source |
| --- | --- | --- |
| pET28a | Vector for protein expression; Kan^r^ | Novagen |
| pME6522 | pVS1-p15A *E. coli*-*S. histidinilytica* shuttle vector for transcriptional *lacZ* fusion and promoter probing, Tet^r^ | Lab stock |
| pET28a-GntR | pET28a with *gntR* gene for expression | This study |
| pTrc99a-*gntR* | pTrc99a with *gntR* gene for overexpression | This study |
| pET28a-GntR_mt_ | pET28a with *gntR_mt_* gene for expression | This study |
| pET28a-XylR | pET28a with *xylR* gene for expression | This study |
| pET28a-XylR_mt_ | pET28a with *xylR_mt_* gene for expression | This study |
| pETRABC | Plasmid pET28a carrying 2,3-BD gene cluster with its operon originated from *E. cloacae* SDM, Kan^r^ | Lab stock |
| pME6522-P*_edd-eda_* | Plasmid pME6522 containing the promoter of gene *edd-eda* | This study |
| pME6522-P*_gntKU_* | Plasmid pME6522 containing the promoter of gene *gntKU* | This study |
| pME6522-P*_xylAB_* | Plasmid pME6522 containing the promoter of gene *xylAB* | This study |
| pME6522-P*_xylFGH_* | Plasmid pME6522 containing the promoter of gene *xylFGH* | This study |
| pEcCas | Vector for constitutive expression of Cas9 and inducible expression of λ-Red recombinase, *sacB*, P*_rhaB_*-sgRNA-pMB1, pSC101, Kan^r^ | Addgene |
| pEcCas_cm_ | Vector for constitutive expression of Cas9 and inducible expression of λ-Red recombinase, *sacB*, P*_rhaB_*-sgRNA-pMB1, pSC101, Cm^r^ | Lab stock |
| pEcgRNA | gRNA expression vector, Spe^r^ | Addgene |
| pEcgRNA-Δ*pgi* | pEcgRNA derivative for transcribing sgRNA to knock out *pgi* | This study |
| pEcgRNA-Δ*gnd* | pEcgRNA derivative for transcribing sgRNA to knock out *gnd* | This study |
| pEcgRNA-Δ*xylA* | pEcgRNA derivative for transcribing sgRNA to knock out *xylA* | This study |
| pEcgRNA-Δ*zwf* | pEcgRNA derivative for transcribing sgRNA to knock out *zwf* | This study |
| pEcgRNA-Δ*ptsG* | pEcgRNA derivative for transcribing sgRNA to knock out *ptsG* | This study |
| pEcgRNA-Δ*cyaA* | pEcgRNA derivative for transcribing sgRNA to replace the *cyaA* with *cyaA_mt_* | This study |
| pEcgRNA-Δ*gntR* | pEcgRNA derivative for transcribing sgRNA to replace the *gntR* with *gntR_mt_* | This study |
| pEcgRNA-Δ*xylR* | pEcgRNA derivative for transcribing sgRNA to replace the *xylR* with *xylR_mt_* | This study |
| pEcgRNA-Δ*CP4-6* | pEcgRNA derivative for transcribing sgRNA to knock out *CP4-6* | This study |
| pEcgRNA-Δ*hfq* | pEcgRNA derivative for transcribing sgRNA to replace the *hfq* with *hfq_mt_* | This study |
| pEcgRNA-Δ*malT* | pEcgRNA derivative for transcribing sgRNA to replace the *malT* with *malT_mt_* | This study |
| pEcgRNA-Δ*poxB* | pEcgRNA derivative for transcribing sgRNA to knock out *poxB* | This study |
| pEcgRNA-Δ*pta*-*ackA* | pEcgRNA derivative for transcribing sgRNA to knock out *pta*-*ackA* | This study |
| pEcgRNA-Δ*ldhA* | pEcgRNA derivative for transcribing sgRNA to knock out *ldhA* | This study |
| pEcgRNA-Δ*frdA* | pEcgRNA derivative for transcribing sgRNA to knock out *frdA* | This study |
| pEcgRNA-Δ*adhE* | pEcgRNA derivative for transcribing sgRNA to knock out *adhE* | This study |
| pEcgRNA-Δ*pflB* | pEcgRNA derivative for transcribing sgRNA to knock out *pflB* | This study |
| PDL-YC-pEcgRNA-Δ*pgi* | pEcgRNA derivative for transcribing sgRNA to knock out *pgi* in *K. oxytoca* | This study |
| PDL-YC-pEcgRNA-Δ*gnd* | pEcgRNA derivative for transcribing sgRNA to knock out *gnd* in *K. oxytoca* | This study |
| PDL-YC-pEcgRNA-Δ*gntR* | pEcgRNA derivative for transcribing sgRNA to replace the *gntR* with *gntR_mt_* in *K. oxytoca* | This study |
| PDL-YC-pEcgRNA-Δ*xylR* | pEcgRNA derivative for transcribing sgRNA to replace the *xylR* with *xylR_mt_* in *K. oxytoca* | This study |

**Table S5**. Primers used in this study

| Primer | Sequence (5′-3′) |
| --- | --- |
| Δ*pgi*-F1 | ttcagccttggcacaagggaagag |
| Δ*pgi*-R1 | cgcgccacgctttatatcattagcaatactcttctgattttgagaat |
| Δ*pgi*-F2 | cagaagagtattgctaatgatataaagcgtggcgcggttaatcat |
| Δ*pgi*-R2 | gccgcaagcgcagatatggcaaaa |
| Δ*gnd*-F1 | atgaaatgactgagtcagccgagaa |
| Δ*gnd*-R1 | ccagccattcggtatgcattacatactcctgtcaggtgtgatc |
| Δ*gnd*-F2 | cctgacaggagtatgtaatgcataccgaatggctggattaatctga |
| Δ*gnd*-R2 | aacgggaaccgttgatttgatgacca |
| Δ*ptsG*-F1 | acgccgtctggcggcagaagagg |
| Δ*ptsG*-R1 | gtcttacggaaattgagagtgctcctgagtatgggtg |
| Δ*ptsG*-F2 | cactctcaatttccgtaagacgttggggagactaaggc |
| Δ*ptsG*-R2 | gagcctggcgcaggacgatacca |
| Δ*zwf*-F1 | GTTCGCTAACATTGGCTTCCAGTGC |
| Δ*zwf*-R1 | TCCAGGAACGACCATCTGTTTGCGTTACCGCCATGTCATTCT |
| Δ*zwf*-F2 | atggcggtaacgcaaacagatggtcgttcctggaatgagtttgagtaa |
| Δ*zwf*-R2 | AGGAGGTGATGATGGCGATATTGTTA |
| Δ*xylA*-F1 | tcttcATTGCCATTTGCAGACTGTAC |
| Δ*xylA*-R1 | gttagccgttatttgtcgaaggcttgcatattgaactccataatcag |
| Δ*xylA*-F2 | tatggagttcaatatgcaagccttcgacaaataacggctaactgtgcag |
| Δ*xylA*-R2 | ccccgtcatacgcagacgcaagtaatc |
| Δ*CP4-6*-F1 | GTATTCGCCATCTTCCACGCGGTTGAGG |
| Δ*CP4-6*-R1 | GGTACGTGAAACATCCGGGCTGAAAATTCGGTGATGCTGCCAACTTACTGATT |
| Δ*CP4-6*-F2 | AAATCAGTAAGTTGGCAGCATCACCGAATTTTCAGCCCGGATGTTTCACGTACC |
| Δ*CP4-6*-R2 | TGACTTCCAGCAGAACTACCAGGCGC |
| Δ*gntR*-F1 | TGCATCTGTATCAGATCTGGATCATG |
| Δ*gntR*-R1 | ACTTCAAATTTGTGGGCCGTCCTGAAGGTACAAAAGAGATAGATT |
| Δ*gntR*-F2 | cttttgtaccttcaggacggcccacaaatttgaagtagctcacac |
| Δ*gntR*-R2 | tttgcgcgctttcaggcggctttca |
| Δ*poxB*-F1 | GCGGCCCGGCTCCGTATATGG |
| Δ*poxB*-R1 | CCTTATTATGACGGGAAATGCCACCCTTTGGTTCTCCATCTCCTGAATGTG |
| Δ*poxB*-F2 | ATCACATTCAGGAGATGGAGAACCAAAGGGTGGCATTTCCCGTCATAATAAGG |
| Δ*poxB*-R2 | AATTCCCATGCTTCTTTCAGGT |
| Δ*pta-ackA*-F1 | CGATCGGCGGCATAAAACGGATCGCATAA |
| Δ*pta-ackA*-R1 | GCAAAGCTGCGGATGATGACGAGAGGAAGTACCTATAATTGATACGT |
| Δ*pta-ackA*-F2 | ACGTATCAATTATAGGTACTTCCTCTCGTCATCATCCGCAGCTTTGC |
| Δ*pta-ackA*-R2 | CGTTAGTCAGAATGCGAAATGAGTGTG |
| Δ*ldhA*-F1 | CAAGCAGAATCAAGTTCTACCATGCCGACGT |
| Δ*ldhA*-R1 | AGGGGAGCGGCAAGAAAGACTTTCTCCAGTGAT |
| Δ*ldhA*-F2 | ACTGGAGAAAGTCTTTCTTGCCGCTCCCCTGC |
| Δ*ldhA*-R2 | TGTCTGTTTCGCGGTCGCCAGCGTTAA |
| Δ*frdA*-F1 | CGTGTCTCAAACGGGACCAAATGAATATCG |
| Δ*frdA*-R1 | TTCAGGTTTTTCATCGACATTCCTCCAGATTGTTTT |
| Δ*frdA*-F2 | ATCTGGAGGAATGTCGATGAAAAACCTGAAAAT |
| Δ*frdA*-R2 | GGTCTGGATGTTAGTACCCTGATCCGCG |
| Δ*adhE*-F1 | AAAATCAAAAAAGGTCTGAATCACGGTTAGCTC |
| Δ*adhE*-R1 | CCAGACAGCGCTACTGAAATGCTCTCCTGATAA |
| Δ*adhE*-F2 | TATCAGGAGAGCATTTCAGTAGCGCTGTCTGGCA |
| Δ*adhE*-R2 | ATTAAAAACCATCTGTTTTTGTGGCCGTAAAG |
| Δ*pflB*-F1 | ATATGACCGCAAATGGTCAATG |
| Δ*pflB*-R1 | ACTGTACGATTTCAGTCAAATCTAAGTAACACCTACCTTCTTAAGTG |
| Δ*pflB*-F2 | TCCACTTAAGAAGGTAGGTGTTACTTAGATTTGACTGAAATCGTACAGT |
| Δ*pflB*-R2 | ACAGGTATGAATGCCTTCTTTTTTGC |
| *cyaA_mt_*-F | GCGGCGAAACCTTCTGGGATTTGCTGGA |
| *cyaA_mt_*-R | GCTGGTAAACCCGCAGATTTCACCCGAT |
| *gntR_mt_*-F | CAGGACGATGAAAAAGAAAAGACCCG |
| *gntR_mt_*-R | TTAAATAGATCCGCCCGGTGACAAGGTGAA |
| *xylR_mt_*-F | ATGTTTACTAAACGTCACCGCATCAC |
| *xylR_mt_*-R | TTCGCTGTAGCGGCATTTCTTCTTT |
| *hfq_mt_*-F | tctcatacgatgaaatggtttatc |
| *hfq_mt_*-R | acaccggcggaagagaccagagatt |
| *malT_mt_*-F | GCATTAGAAAGGTTTCTGGCCGAC |
| *malT_mt_*-R | AGATGATAGTCATCGATGACCAGAT |
| P*_xylFGH_*-F | ATATTGAACTCCATAATCAGGTAATGC |
| P*_xylFGH_*-R | CGCAATTGTACTTATTGCATTTTTCTC |
| P*_xylAB_*-F | GAGAAAAATGCAATAAGTACAATTGCG |
| P*_xylAB_*-R | CATGGTGTAGGGCCTTCTGT |
| P*_edd-eda_*-F | TATCTGCGCTTATCCTTTATGGTT |
| P*_edd-eda_*-R | AAAGGCTCCTGAAATTGAGTTG |
| P*_gntKU_*-F | GCCCACAAATTTGAAGTAGCTCAC |
| P*_gntKU_*-R | AGTGGTGCCCCCACAATACAAGAA |
| P*_gntT_*-F | CGGAATTCCCGGAGCTGAAAGGTGTGCGCG |
| P*_gntT_*-R | CCGATAGCAACAATGACTAAT |
| GntR-F | CCGGAATTCATGAAAAAGAAAAGACCCGTACT |
| GntR-R | CCCAAGCTTTTAAATAGATCCGCCCGGTG |
| XylR-F | ctggtgccgcgcggcagccaTATGTTTACTAAACGTCACCGCATC |
| XylR-R | cgggctttgttagcagccggaCTACAACATGACCTCGCTATTTACA |
| pME6522-P*_edd-eda_*-F | CGGAATTCTATCTGCGCTTATCCTTTATGGTT |
| pME6522-P*_edd-eda_*-R | AACTGCAGAAAGGCTCCTGAAATTGAGTTG |
| pME6522-P*_gntKU_*-F | CGGAATTCGCCCACAAATTTGAAGTAGCTCAC |
| pME6522-P*_gntKU_*-R | AACTGCAGAGTGGTGCCCCCACAATACAAGAA |
| pTrc99a-*gntR*-F | AACAGACCATGGAATTCatgaaaaagaaaagacccgtacttc |
| pTrc99a-*gntR*-R | ATCCGCCAAAACAGCCAAGCTTttaaatagatccgcccggtgacaaggt |
| pME6522-P*_xylAB_*-F | CGGAATTCGGTGTAGGGCCTTCTGTAGTT |
| pME6522-P*_xylAB_*-R | AACTGCAGATTGAACTCCATAATCAGGT |
| pME6522-P*_xylFGH_*-F | CGGAATTCATTGAACTCCATAATCAGGT |
| pME6522-P*_xylFGH_*-R | AAAACTGCAGGGTGTAGGGCCTTCTGTAGTTAG |
| ***K. oxytoca* PDL-YC** |  |
| Δ*pgi*-F1 | gcttcgcgaaacgttcgccgggttc |
| Δ*pgi*-R1 | cattgcgaacgttttagcgatagcaatactcttctgatttgagaa |
| Δ*pgi*-F2 | caaatcagaagagtattgctatcgctaaaacgttcgcaatgccggc |
| Δ*pgi*-R2 | ccaatgccagctatcgccaccactt |
| Δ*gnd*-F1 | cgcggctttttttaaagctcgagc |
| Δ*gnd*-R1 | ggcctgactgcgtcaagaagttacatactcctgtcatatgtgtaaa |
| Δ*gnd*-F2 | cacatatgacaggagtatgtaacttcttgacgcagtcaggccatttg |
| Δ*gnd*-R2 | gccgctatcggccgcattgaccgca |
| *gntR_mt_*-F1 | atcgcagtcaggacgATGAAAAAGA |
| *gntR_mt_*-R1 | GATAGTACGTTCATCAAGATAGGCAACGTGACGATGGCCAT |
| *gntR_mt_*-F2 | ACGTTGCCTATCTTGATGAACGTACTATCATCAAACAGA |
| *gntR_mt_*-R2 | TTAAATAGATCCGCCCGGTGACAAT |
| *xylR_mt_*-F1 | atgtttgaaaagcgtcaccgtattac |
| *xylR_mt_*-R1 | gggcaggccgtaaaaggcgaagctgttaatgcctttttccttcagatg |
| *xylR_mt_*-F2 | ggaaaaaggcattaacagcttcgccttttacggcctgcccgcttccagc |
| *xylR_mt_*-R2 | cgacggatagccgcacatctggga |

**REFERENCES**

1. Dev C, Jilani SB, Yazdani SS. 2022. Adaptation on xylose improves glucose–xylose co-utilization and ethanol production in a carbon catabolite repression (CCR) compromised ethanologenic strain. Microb Cell Fact 21:154.

2. Fujiwara R, Noda S, Tanaka T, Kondo A. 2019. Metabolic engineering of *Escherichia coli* for shikimate pathway derivative production from glucose-xylose co-substrate. Nat Commun 10:4559.

3. Sievert C, Nieves LM, Panyon LA, Loeffler T, Morris C, Cartwright RA, Wang X. 2017. Experimental evolution reveals an effective avenue to release catabolite repression via mutations in XylR. Proc Natl Acad Sci U S A 114:7349–7354.

4. Martinez R, Flores AD, Dufault ME, Wang X. 2019. The XylR variant (R121C and P363S) releases arabinose-induced catabolite repression on xylose fermentation and enhances coutilization of lignocellulosic sugar mixtures. Biotechnol Bioeng 116:3476–3481.

5. Gawand P, Hyland P, Ekins A, Martin VJJ, Mahadevan R. 2013. Novel approach to engineer strains for simultaneous sugar utilization. Metab Eng 20:63–72.

6. Zhao C, Sinumvayo JP, Zhang Y, Li Y. 2019. Design and development of a “Y-shaped” microbial consortium capable of simultaneously utilizing biomass sugars for efficient production of butanol. Metab Eng 55:111–119.
